# Pathway-Based Kernel Boosting for the Analysis of Genome-Wide Association Studies

**DOI:** 10.1155/2017/6742763

**Published:** 2017-07-13

**Authors:** Stefanie Friedrichs, Juliane Manitz, Patricia Burger, Christopher I. Amos, Angela Risch, Jenny Chang-Claude, Heinz-Erich Wichmann, Thomas Kneib, Heike Bickeböller, Benjamin Hofner

**Affiliations:** ^1^Institute of Genetic Epidemiology, University Medical Centre, Georg-August University Göttingen, Göttingen, Germany; ^2^Department of Statistics and Econometrics, Georg-August University Göttingen, Göttingen, Germany; ^3^Department of Mathematics and Statistics, Boston University, Boston, MA, USA; ^4^Department of Community and Family Medicine, Geisel School of Medicine, Dartmouth College, Lebanon, NH, USA; ^5^Division of Molecular Biology, University of Salzburg, Salzburg, Austria; ^6^Translational Lung Research Center Heidelberg (TLRC-H), Member of the German Center for Lung Research (DZL), Heidelberg, Germany; ^7^Division of Epigenomics and Cancer Risk Factors, German Cancer Research Center (DKFZ), Heidelberg, Germany; ^8^Division of Cancer Epidemiology, German Cancer Research Center (DKFZ), Heidelberg, Germany; ^9^Institute of Medical Informatics, Biometry and Epidemiology, Chair of Epidemiology, Ludwig-Maximilians University, Munich, Germany; ^10^Helmholtz Center Munich, Institute of Epidemiology II, Munich, Germany; ^11^Institute of Medical Statistics and Epidemiology, Technical University Munich, Munich, Germany; ^12^Department of Medical Informatics, Biometry and Epidemiology, Friedrich-Alexander-Universität Erlangen-Nürnberg, Erlangen, Germany; ^13^Section Biostatistics, Paul-Ehrlich-Institut, Langen, Germany

## Abstract

The analysis of genome-wide association studies (GWAS) benefits from the investigation of biologically meaningful gene sets, such as gene-interaction networks (pathways). We propose an extension to a successful kernel-based pathway analysis approach by integrating kernel functions into a powerful algorithmic framework for variable selection, to enable investigation of multiple pathways simultaneously. We employ genetic similarity kernels from the logistic kernel machine test (LKMT) as base-learners in a boosting algorithm. A model to explain case-control status is created iteratively by selecting pathways that improve its prediction ability. We evaluated our method in simulation studies adopting 50 pathways for different sample sizes and genetic effect strengths. Additionally, we included an exemplary application of kernel boosting to a rheumatoid arthritis and a lung cancer dataset. Simulations indicate that kernel boosting outperforms the LKMT in certain genetic scenarios. Applications to GWAS data on rheumatoid arthritis and lung cancer resulted in sparse models which were based on pathways interpretable in a clinical sense. Kernel boosting is highly flexible in terms of considered variables and overcomes the problem of multiple testing. Additionally, it enables the prediction of clinical outcomes. Thus, kernel boosting constitutes a new, powerful tool in the analysis of GWAS data and towards the understanding of biological processes involved in disease susceptibility.

## 1. Introduction

Many human diseases are complex in nature. They are caused by an interplay of several, often moderate genetic effects and environmental factors (i.e., demographic, clinical, and other nongenetic data [1]). Their genetic architecture is often analyzed in genome-wide association studies (GWAS). Herein, genetic information is represented by the genotypes of a multitude of single-nucleotide polymorphisms (SNPs) located across the whole genome. Numerous SNPs associated with various diseases have already been discovered in GWAS analyses; however they cannot account for the full heritability of the corresponding disease [2]. Different methods to approach this problem of* missing heritability* have been proposed, including the joint analysis of several SNPs representing a particular part of the genetic information, such as a gene or gene set.

Gene-set analysis methods facilitate the detection of associations between an individual's genetic information and a phenotype of interest, for example, disease status. The joint analysis of several genes often leads to increased power, as it reduces the overall number of conducted tests and assists in the detection of moderate associations [3]. Furthermore, the results are usually more meaningful, as they are based on functional units rather than on single SNPs. One form of gene-set analysis is the investigation of pathways, such as networks of interacting genes responsible for a specific cell function or regulation [4]. The proteins coded by genes within a pathway can enhance or reduce the expression of other genes, to which we refer as activation or inhibition. Thus, genes interact directly as well as indirectly in a series of interconnected steps within pathways. Different types of biological pathway exist, for example, involved in metabolism or signal transduction. Faults in function can occur and such malfunction of biological pathways may lead to disease onset and development.

Large sample sizes are required to detect weak genetic effects influencing disease risk. Thanks to technical advances and the formation of data-sharing consortia in particular, larger GWAS datasets have become available over recent years. However, genotyping and participant recruitment are still cost and work intensive. Especially in rare diseases, taking as an example the analysis of histological subtypes of a disease, it is very challenging to achieve sample sizes that result in adequate power in analyses [5]. Another challenge we face is to understand the biological meaning of detected associations. It is often difficult to interpret the results of GWAS analysis in the elucidation of the precise biological processes and corresponding functional units influencing disease susceptibility. Single-pathway analysis methods are often successful in the identification of genetic effects influencing disease susceptibility. However, they usually can not discriminate causal biological processes from isolated effects included in pathways due to gene overlap [6, 7]. Another limitation of many pathway analysis approaches is the lacking ability to predict the disease state, or other outcomes of interest, based on the identified genetic effects.

Kernel methods in statistics have already been demonstrated as dealing well with the challenges faced when analyzing GWAS data [8, 9]. They are capable of handling high-dimensional data, without requiring any direct specification of the functional relationship between genetic effects. Furthermore, kernel methods are computationally efficient and allow the straightforward incorporation of environmental covariates [9–11]. Kernels are used to calculate a quantitative value from genotype data, which may be interpreted as reflecting the genetic similarity between each pair of individuals. Different kernels have been proposed in the analysis of pathways [9, 12, 13]. While some kernels only evaluate SNP membership in genes, others can also adjust for differing gene numbers and sizes or even include gene interaction structures or other information (please refer to Materials and Methods and [13] for an overview). We focussed on the network-based kernel, as it allows us to include interaction structures and has been demonstrated as being superior in performance for interconnected effects [13].

We extend kernel-based analysis of GWAS data by integrating a network-based kernel function into a boosting framework, in order to identify genetic variation modulating disease susceptibility. Boosting emerged from the field of machine learning and was later transferred to statistical modelling. It implements an ensemble of many weak learners (so-called base-learners, simple models that are slightly improved over random guessing) to optimize the predictive accuracy of a model [14]. Since it is able to combine the power from several predictors with weak signals into a strong prediction set [15, 16], it may prove to be a powerful tool in the analysis of GWAS. Component-wise boosting enforces variable selection and includes additional effect regularization, which makes it especially useful for high-dimensional data [17]. Model-based boosting can be seen as an extension of classic boosting approaches (see, e.g., [18, 19]). Diverse base-learners, which represent special effect types, may be chosen and combined arbitrarily [20]. Thus, boosting allows the simultaneous inclusion of genetic information and demographic or other environmental data. This joint investigation of multiple variables allows taking into account correlations between different pathways and will likely facilitate discrimination of causal biological processes from effects included in pathways only due to gene overlap. The derived models can be assessed and interpreted directly. Our kernel boosting approach overcomes the problem of multiple testing thanks to its inherent variable selection property [21]. Thereby the overall gain in power in the analysis of GWAS supports the analysis of smaller samples and moderate-to-weak genetic effects. Of note, the main focus of boosting (as well as of other machine learning methods) is not on hypothesis testing but on the development of a multivariable prediction model.

We applied our approach to two GWAS datasets, one on lung cancer and one on rheumatoid arthritis. Lung cancer is one of the most common forms of cancer, especially in industrialized nations. It is responsible for the greatest proportion of deaths caused by cancer worldwide [22]. Although the exposure to tobacco is known to be the major risk factor for lung cancer susceptibility, a number of genetic influences have been revealed by many studies [23]. The actual number of known genetic influences, excepting some specific lung cancer syndromes, is still limited, and each only accounts for a minor increase in disease risk. Rheumatoid arthritis is the most frequently occurring inflammatory disease of the joints, predominantly affecting the hands and feet. It is one of the major causes of disability and is strongly influenced by genetic factors in the human leukocyte antigen (HLA) region located on chromosome 6 [24, 25]. The investigation into these two diseases with different genetic architectures provides the ideal platform to evaluate the performance of our novel method.

In Section 2, we introduce the model structure utilized and describe the construction of network-based kernel functions. We provide a short introduction to boosting and derive the novel boosting algorithm with kernel-based base-learners.  Section 3 comprises a description of the simulation study used to evaluate the method's performance and an overview of the application to rheumatoid arthritis and lung cancer GWAS datasets. The results of the simulation study and GWAS analyses are summarized in Section 4. Finally, we end the paper with a discussion and an outlook.

### 1.1. Software

We used the statistical software environment R [26] to perform all analyses unless stated otherwise. The methodological developments were implemented in the R packages kangar00 [27] and mboost [28]. An exemplary application of the kernel boosting method to a simulated data set is given in Supplementary Material 2, available online at https://doi.org/10.1155/2017/6742763.

## 2. Materials and Methods

We aim to model the disease status of an individual, based on environmental covariates and genetic information obtained from GWAS. The genetic information given by the genotypes of different SNPs is mapped via genes to pathways. For each pathway, we compute a kernel matrix transforming the genotype vectors of each two individuals into a numeric value, which may be interpreted as the genetic similarity of the two individuals. Based on these matrices, we fit a kernel-based boosting model to identify relevant pathways and to find a prediction model for disease status. In the following paragraphs, we define all the relevant parts to this approach.

### 2.1. Model Definition and Notation

We assume an additive logistic regression model for the conditional probability of being a case for individual *i*, *i* = 1,…, *n*:

(1) $$\begin{array}{c} \mathrm{logit}\left[\;P\left(\;y_{i}=1\;|\;x_{i},z_{i}\;\right)\;\right]=\eta \left(\;x_{i},z_{i}\;\right), \end{array}$$

with additive predictor

(2) $$\begin{array}{c} \eta \left(\;x_{i},z_{i}\;\right)=x_{i}\beta +f_{1}\left(\;z_{i}\;\right)+\cdots +f_{P}\left(\;z_{i}\;\right), \end{array}$$

where *y*_*i*_ is the case-control indicator (*y*_*i*_ = 0 control; *y*_*i*_ = 1 case), **x**_*i*_ = (*x*_*i*,1_,…, *x*_*i*,*n*_*c*__) is the *n*_*c*_ dimensional environmental covariate vector, and **z**_*i*_ denotes the genotype vector of the *n*_*s*_ SNPs of the *i*th individual. Note that the non- or semiparametrically modelled genetic effects *f*_*p*_(**z**_*i*_) usually only depend on a pathway specific subset of SNPs, **z**_*i*_^(*p*)^. However, for the sake of notational convenience we dropped the pathway index (*p*).

The vector **β** = (*β*_0_, *β*_1_,…, *β*_*n*_*c*__)^*⊤*^ represents the regression coefficients (including an intercept *β*_0_) related to the environmental covariates. They typically include information on age, sex, or other traits relevant to the disease investigated. The genotype variables **z**_*i*_ are coded as number of minor alleles, resulting in *z*_*i*,*s*_ ∈ {0,1, 2} for any SNP *s* and individual *i*. The nonparametric functions *f*_*p*_, *p* = 1,…, *P*, describe how the risk of being affected by the disease depends on the observed genotypes. Here, we aggregate the genotype information according to SNP membership in *P* different gene interaction pathways.

### 2.2. Network-Based Kernels

Liu et al. [10] introduced the kernel machine framework to the field of pathway analysis. Since genes in pathways can include complex interactions, nonparametric approaches are advisable. The logistic kernel machine test (LKMT) can model the effect of a pathway on a binary outcome nonparametrically, while including parametrically modelled covariates. In the resulting logistic regression model, the genetic influence is incorporated by a function from the reproducing kernel Hilbert space generated by a positive definite kernel function *K*.

In a genetic application, this kernel function is evaluated for the genotypes of each two individuals *i* and *j*, whereby the kernel matrix element *K*_*ij*_ = *K*(**z**_*i*_, **z**_*j*_) is obtained. This value can be understood as the genetic similarity between the two individuals. To embed this definition into the mathematically well-defined framework of a reproducing kernel Hilbert space, the kernel matrix has to fulfill some requirements: it has to be quadratic, symmetric, and positive semidefinite. A variety of kernel functions are available. In the pathway-based analysis of GWAS data, a network-based kernel can be used, which is able to incorporate the pathway topology [13].

**Figure 1:**
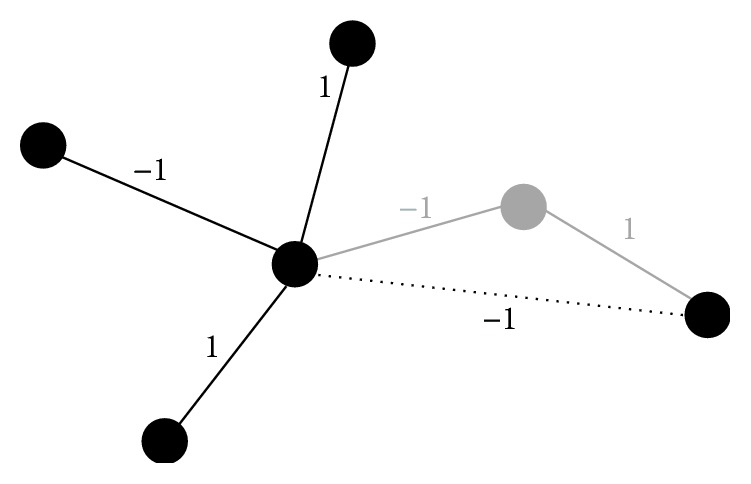
Graphical representation of rewiring step in data preparation. Nodes are representing genes in the pathway, while edges indicate interactions between the corresponding genes. Assume the gene depicted in grey is not represented by any genetic markers in the considered study and thus cannot be analyzed. To retain information about the (indirect) interaction of the two genes previously linked to the omitted gene, a new direct link is established between them. Its interaction type is determined by multiplication of the weights inherent to the two dropped links.

Assume **Z** = (**z**_1_,…, **z**_*n*_)^*⊤*^ denotes the *n* × *n*_*s*_ pathway specific genotype matrix consisting of the genotype vectors **z**_*i*_, which include only the SNPs relevant for pathway *p*, for all *i* = 1,…, *n* individuals. Then, the network-based kernel is defined by

(3) $$\begin{array}{c} K=ZANA^{⊤}Z^{⊤}, \end{array}$$

where **A** is an *n*_*s*_ × *n*_*g*_ matrix mapping all SNPs to the *n*_*g*_ investigated genes (including an adjustment to account for differing sizes of genes) and **N** represents the (modified) *n*_*g*_ × *n*_*g*_ matrix network adjacency matrix of gene interactions. To ensure positive semidefiniteness of the kernel, the network adjacency matrix is processed in a number of preparatory steps: if a gene is not represented by any SNPs in the investigated GWAS dataset, it cannot be considered in the analysis. To prevent loss of information about interactions in the network, genes which have previously been connected via the omitted gene will be linked directly. The new link's weight is determined in a multiplicative fashion, based on the weights of the two omitted links. For a graphical representation refer to Figure 1. The resulting matrix is further mirrored along its diagonal and transformed to obtain positive semidefiniteness. The applied transformation is given by

(4) $$\begin{array}{c} \rho N+\left(\;1-\rho \;\right)I, \end{array}$$

where **I** denotes the identity matrix and *ρ* is a weight based on the smallest eigenvalue of **N**. For more details, see [13].

### 2.3. Model-Based Boosting

Model fitting in general aims to minimize the loss when relating observed responses *y*_*i*_ to an estimated model characterized by the additive predictor *η*_*i*_≔*η*(**x**_*i*_, **z**_*i*_) as defined in (2). Thus, boosting minimizes the empirical risk

(5) $$\begin{array}{c} \frac{1}{n}\overset{n}{\underset{i=1}{\sum }}-l\left(\;y_{i},\eta_{i}\;\right), \end{array}$$

where −*l*(·) denotes a suitable loss function. Here, we use the negative binomial log-likelihood as loss function, which results in additive logistic regression models in analogy to the LKMT. In general, the loss function characterizes the model and can be defined in terms of a suitable negative log-likelihood or other appropriate loss functions, for example, the quadratic error loss for Gaussian regression or the absolute error loss for quantile regression. For an overview on loss functions see Hofner et al. [20]. Boosting solves this optimization problem via functional gradient descent by moving in the direction of the loss function's steepest descent along the additive effects of predictor (2). This can be seen in the following (simplified) algorithm:

(1) Initialize the additive predictor with ${{\hat{\eta }}_{i}}^{\left[0\right]}=\bar{y},\;\;i=1,\ldots ,n$, and all function estimates with ${\hat{f}}_{p}^{\left[0\right]}=0,\;\;p=1,\ldots ,P^{+}$. Note that *P*^+^ includes all *P* kernels and possibly additional effects for environmental covariates.
(2) For *m* = 1,…, *m*_stop_ do the following:
  (a) Compute the negative gradient of the loss function evaluated at the estimates of the previous iteration:
    (6) $$\begin{array}{c} u_{i}^{\left[m\right]}=-{\left.\;\frac{\partial \left(-l\left(y_{i},\eta_{i}\right)\right)}{\partial \eta }\;\right|}_{\eta_{i}={\hat{\eta }}^{\left[m-1\right]}\left(x_{i},z_{i}\right)},\;i=1,\ldots ,n. \end{array}$$
  (b) Estimate the negative gradient vector **u**^[*m*]^ = (*u*_1_^[*m*]^,…, *u*_*n*_^[*m*]^)* separately* for each effect in the additive predictor (2) by base-learners ${\hat{u}}^{\left[m\right]}={\hat{f}}_{p},\;\;p=1,\ldots ,P^{+}$, with ${\hat{f}}_{p}≔{\left({\hat{f}}_{p}(x_{i},z_{i})\right)}_{i=1,\ldots ,n}$ by fitting simple regression models via (penalized) least squares. Thus, each base-learner regresses the negative gradient vector **u**^[*m*]^ separately on each of the predictors.
  (c) Choose the best-fitting base-learner ${\hat{f}}_{p^{⋆}}$ with the minimal residual sum of squares.
  (d) Compute the update for the additive predictor by adding the best-fitting base-learner with a step-length factor 0 < *ν* ≤ 1:
    (7) $$\begin{array}{c} {\hat{\eta }}^{\left[m\right]}={\hat{\eta }}^{\left[m-1\right]}+\nu \cdot {\hat{f}}_{p^{⋆}}. \end{array}$$
  The corresponding update of function estimate ${\hat{f}}_{p^{⋆}}$ is given by
    (8) $$\begin{array}{c} {\hat{f}}_{p^{⋆}}^{\left[m\right]}={\hat{f}}_{p^{⋆}}^{\left[m-1\right]}+\nu \cdot {\hat{f}}_{p^{⋆}}, \end{array}$$
  while
    (9) $$\begin{array}{c} {\hat{f}}_{p}^{\left[m\right]}={\hat{f}}_{p}^{\left[m-1\right]}, \end{array}$$
  for all *p* ≠ *p*^⋆^.

**Figure 2:**
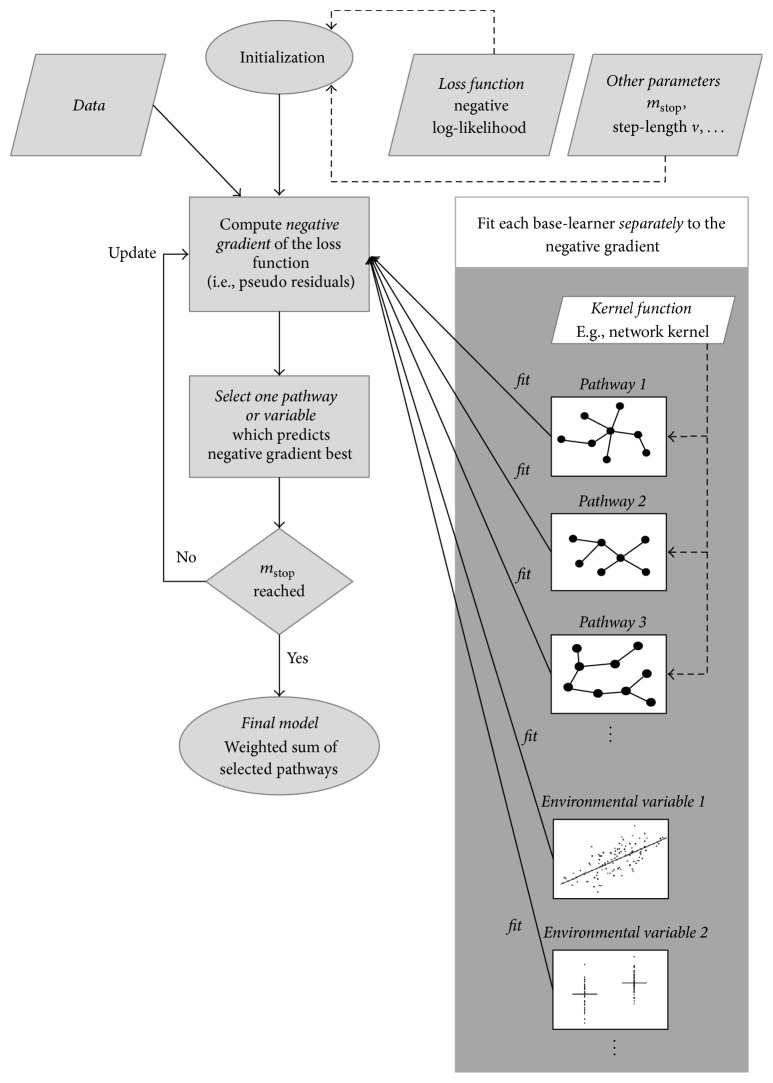
Graphical representation of the main features of the kernel boosting algorithm.

Note that each base-learner ${\hat{f}}_{p}$ usually depends on only one environmental covariate or one pathway based on a suitable subset of the genotypes of **z**. However, other dependencies are also possible. For details on the algorithm, see [20]. A graphical display of the main features of the kernel boosting algorithm is given in Figure 2.

### 2.4. Model Tuning

The major tuning parameter of the functional gradient descent boosting algorithm is the number of iterations *m*_stop_. We usually choose *m*_stop_ via cross-validation methods (such as bootstrap, *k*-fold cross-validation, or subsampling) in order to avoid overfitting: one fits the model on the selected subset of the data and chooses *m*_stop_ such that it minimizes the empirical risk on the data that were not used to estimate the model. Subsampling is recommended to avoid overly complex models [29]. The step-length *ν* is another tuning parameter. In general it is of minor importance as long as it is relatively small. It determines the trade-off between speed of convergence and variable selection ability and is typically set to 0.1 [30].

The current estimate ${\hat{\eta }}^{\left[m\right]}$ of the additive predictor **η** usually depends on only a subset of the possible predictors: as we select the best-fitting base-learner in each step and choose *m*_stop_ such that it maximizes prediction accuracy (i.e., usually relatively small so that not all base-learners are selected), boosting selects base-learners and thus variables. In our approach, we exploit this behaviour to identify genetic associations. Note that a base-learner can be selected multiple times. Hence, its function estimate ${\hat{f}}_{p},\;\;p\in 1,\ldots ,P^{+}$, is the weighted sum with weights *ν* of the individual estimates over all iterations in which the base-learner was selected (see (8)).

### 2.5. Boosting with Network-Based Kernel as Base-Learner

To incorporate genotype data, aggregated to represent a particular pathway, we utilize kernel-based base-learners. Using a kernel function *K*, we transform the definition of the genotypic information of all pairs of individuals to *K*_*ij*_ = *K*(**z**_*i*_, **z**_*j*_), *i*, *j* = 1,…, *n*, as mentioned before, and collect them in the kernel matrix **K**. With this matrix, we can estimate

(10) $$\begin{array}{c} f\left(\;Z\;\right)=K\gamma =ZANA^{⊤}Z^{⊤}\gamma , \end{array}$$

The function *f*(**Z**) is used to map the influence of SNP profiles to the clinical outcome (see (2)). As we expect patients with similar SNP profiles to have similar outcomes, we aim to discourage large differences in *f*(**Z**) for genetically similar individuals. According to the standard penalization approaches in the boosting context, we thus introduce an additional smoothness constraint on the coefficient vector **γ** = (*γ*_1_,…, *γ*_*n*_)^*⊤*^ based on the kernel distances:

(11) $$\begin{array}{c} J\left(\;\gamma \;\right)=\gamma^{⊤}K\gamma . \end{array}$$

Thus, we define a separate kernel base-learner for each pathway in the boosting framework. Using the negative gradient vector **u**^[*m*]^ = (*u*_1_^[*m*]^,…, *u*_*n*_^[*m*]^) from the *m*th boosting iteration, we can estimate the coefficient vector **γ** of each base-learner (see step 2b of the algorithm) via penalized least squares

(12) $$\begin{array}{c} {\hat{\gamma }}^{\left[m\right]}={\left(\;K^{⊤}K+\lambda K\;\right)}^{-1}K^{⊤}u^{\left[m\right]}, \end{array}$$

where we dropped the function index *p* for the sake of notational convenience. Note that kernel matrix **K** plays the role of design matrix as well as the role of penalty matrix with penalty parameter *λ*, which governs the smoothness of the estimate. Usually, the penalty parameter *λ* is chosen such that all base-learners have equal degrees of freedom to allow an unbiased selection. A common choice is four degrees of freedom if only smooth effects are used or one degree of freedom if linear effects are to be included; see Hofner et al. [21] for details.

In some rare cases, the derived kernel matrix **K** is numerically not positive semidefinite (i.e., minimal deviations might occur), even though this should theoretically always be the case. To ensure a numerically positive semidefinite matrix **K**, we apply transformation (4) not only to **N** but also on the resulting kernel matrix **K**. The proposed approach is very fast and results in smaller absolute differences in the matrix elements than alternatives such as the procedure suggested by Higham [31] (results not shown).

For numerical reasons, we reformulate the estimation problem from (12) by multiplying the design matrix with the inverse of the square root of the penalty matrix [32]. Thus, we obtain the design matrix

(13) $$\begin{array}{c} \tilde{K}=KK^{-1/2}, \end{array}$$

while the penalty matrix simplifies to the identity matrix **I**. Now, we can equivalently write

(14) $$\begin{array}{c} {\hat{\gamma }}^{\left[m\right]}={\left(\;{\tilde{K}}^{⊤}\tilde{K}+\lambda I\;\right)}^{-1}{\tilde{K}}^{⊤}u^{\left[m\right]}. \end{array}$$

A similar approach based on radial basis functions, which, for example, uses correlation functions to measure distances, was introduced to the boosting framework by Hofner [33].

### 2.6. Model Prediction Using Kernels

Boosting specifically aims to optimize prediction accuracy. As in all regression models, we can use the estimated coefficients to predict the outcome for new observations. However, some extra work is required to set up the kernel, that is, the design matrix, with new genotype data **Z**^*∗*^ = (**z**_1_^*∗*^,…, **z**_*n*^⋆^_^*∗*^)^*⊤*^. In this context, the kernel can be understood to compute the similarity between genotype information of individuals to be predicted and the observations used to fit the model, the training data **Z** itself. Thus,

(15) $$\begin{array}{c} K^{⋆}={\left(\;K\left(\;z_{i}^{⋆},z_{j}\;\right)\;\right)}_{i=1,\ldots ,n^{⋆},\;\;j=1,\ldots ,n}=Z^{⋆}ANA^{⊤}Z^{⊤}. \end{array}$$

The resulting kernel **K**^*∗*^ has the dimension *n*^*∗*^ × *n*, with *n*^*∗*^ being new and *n* previously used observations. Note that kernel matrix **K**^*∗*^ must no longer be of full rank nor be positive semidefinite. Using **K**^*∗*^, we can predict the effect of a pathway on the outcome as

(16) $$\begin{array}{c} \hat{f}\left(\;Z^{*}\;\right)=K^{*}\hat{\gamma }, \end{array}$$

where $\hat{\gamma }$ is obtained as the weighted sum with weights *ν* over the estimates from (14) for all iterations in which the *p*th base-learner was selected (see (8)).

### 2.7. Incorporation of Environmental Covariates

To incorporate environmental variables into the boosting model, we can choose different base-learners suited to different types of effect. Linear effect base-learners are suited to a continuous covariate *x* such as patient age, while categorical effect base-learners facilitate the incorporation of categorical environmental variables such as gender. For details on inclusion of environmental variables, refer to [20].

With the inclusion of environmental variables as base-learners, these are also subject to the selection process inherent to boosting and compete with the pathway-based genetic effects. However, one usually wishes to consider only the added effect of genetic pathways. To ascertain that the model is corrected for environmental variables, one may include them as mandatory effects. This can be done by fitting a standard logistic regression model for the effect of the environmental variables on the clinical outcome and using the estimates as a start model (offset) for the boosting algorithm (see [34, 35]). This approach is very similar to the LKMT procedure, which tests if the logistic regression model can be improved via addition of a nonparametric effect incorporating a particular pathway.

## 3. Simulations and Applications

### 3.1. Simulation Study

**Figure 3:**
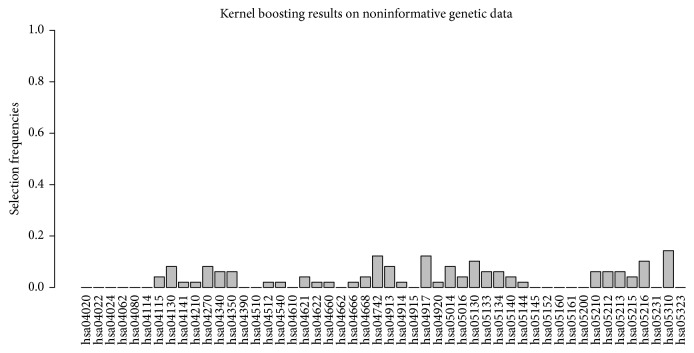
Relative frequency of datasets in which a pathway was selected for 50 pathways in the noninformative simulation scenario.

To evaluate the performance of kernel boosting, we conducted a simulation study based on simulated SNP data in combination with gene networks from existing biological pathways. Pathway information was extracted from the Kyoto Encyclopedia of Genes and Genomes (KEGG) [36]. For simulation purposes, we considered a sample of 50 networks, randomly chosen from the total of 284 pathways available in January 2015. Please refer to Figure 3 for a list of these pathways and refer to Table 1 for their network topology characteristics. The primary aim of this study was to determine whether kernel boosting can detect associated pathways and is able to distinguish them from noninfluential pathways. Thus, we investigated the method's performance on data without genetic effects (null case) including 1000 individuals and in six effect scenarios, differing in effect strengths (relative risk of 1.1 and 1.5 per allele) and sample sizes (*n* ∈ {500,1000,2000} with a 1 : 1 ratio of cases to controls). Datasets for all scenarios were simulated for 100 replications. Note that these scenarios are small compared to typically available sample sizes nowadays. The reason can be found in the computational demands of the method for an insightful number of replications. Accordingly, comparably strong effects of markers were chosen to match the sample sizes used in our simulations.

For each simulated dataset, we fitted a boosting model with pathway kernels. In order to tune the model, that is, to derive the optimal number of boosting steps *m*_stop_, we used 20-fold subsampling for each model on each of the datasets with a maximum number of 200 iterations. Using the network-based kernel function in both methods, we compared the results from our kernel boosting approach on multiple pathways to those obtained from the single-pathway LKMT [9–11]. Additional simulations with cross-validated models and a maximum number of up to 1000 iterations were conducted to gain more insight into the proposed algorithm and are presented in Supplementary Material 1, Section A.

All genotypes were simulated with the help of a reference dataset from the International HapMap Consortium [37]. The reference data include 1,184 individuals of European descent (CEU) and a total of 1,440,616 SNPs, of which 116,565 are located on chromosome one. For each gene included in at least one of the 50 selected pathways, we defined a* pseudogene* to represent the gene within our simulations. Such a* pseudogene* was a randomly selected DNA segment on chromosome one of the reference data including five different SNPs. Between each two sampled regions, we ensured a distance of at least 100 kilo base pairs to prevent distortive LD correlations between them [38]. The location of* pseudogenes* was left unchanged for all simulations, resulting in a realistic correlation structure for all simulation scenarios. In each of the 100 simulation runs, new genotype data for a total of 11,665 SNPs in 2,333* pseudogenes* were simulated using the HAPGEN2 software. This software generates new haplotype data by combining a given set of reference haplotypes with previously simulated data. The detailed procedure is described in [39].

In the null case, noninformative genetic data were simulated for 1000 individuals. In each replication, new genotypes without association signals were generated for 11,665 SNPs. The disease status was assigned at random with 0.5 binomial probability of being a case, completely independent of genotype information. In each of the six effect scenarios, genotype data for a previously chosen equal number of cases and controls were simulated such that two pathways affected disease status. Association signals were included in three genes per causal pathway. In each of the resulting six genes, two randomly selected SNPs were chosen to be influential on the binary clinical outcome. Within one simulation scenario, all associated SNPs had the same effect strength and for each SNP the minor allele was influential. All effects were simulated as additive. To simplify the evaluation, we decided not to include environmental variables in these settings.

We chose two typical pathways (KEGG ids* hsa04020* and* hsa04022*) to include causal genes. In accordance with the findings in [13], the influential genes in the two causal pathways were chosen to be interconnected within the corresponding pathway. Here, we additionally sampled one effect gene in each pathway, with the probability of being selected set to its betweenness centrality. Betweenness centrality measures the amount of shortest connections between each two genes in the network passing through the gene. Different studies have indicated that genes in topologically relevant positions of a pathway are more likely to be involved in disease association [40]. Two neighbouring genes of the sampled gene were randomly chosen to complete the connected scenario. In hsa04020, the genes GNA11, TACR1, and BDKRB2 were simulated to include SNPs influencing disease susceptibility. For hsa04022, genetic effects were placed on the genes PRKG2, ATP2B2, and KCNU1. For each of these genes, two SNPs were simulated as being influential on disease status. Note that existing biological pathways can have genes in common. Thus, beside our two pathways chosen to include influential effects, six additional pathways contain association signals. Refer to Table 2 for an overview of influential genes included in simulation pathways.

*Application: GWAS for Rheumatoid Arthritis and Lung Cancer*. We considered the German Lung Cancer study (GLC) with 488 cases and 478 controls, based on the data of participants taken from the following three individual studies: Lung Cancer in the Young (LUCY), a population-based multicentre study run by the Helmholtz Zentrum Munich, and the University Medical Centre of the Georg-August-University in Goettingen. This study includes data of lung cancer patients under the age of 51 and family members recruited in German hospitals [41, 42]. The Heidelberg lung cancer case-control study, conducted by the German Cancer Research Centre (DKFZ) and the Thoraxklinik in Heidelberg, Germany, recruited cases and controls in a hospital-based study [43]. Additional controls were provided by Cooperative Health Research in the Augsburg Region (KORA), a population-based genome-wide study carried out by the Helmholtz Zentrum Munich [44]. A subset of the study participants of these three studies was chosen to form the German Lung Cancer GWAS. These individuals were genotyped on a HumanHap 550K SNP chip.

The second GWAS is a rheumatoid arthritis study of the North American Rheumatoid Arthritis Consortium (NARAC). It includes 868 cases from New York hospitals, in which rheumatoid arthritis was diagnosed based on the criteria of the American College of Rheumatology. Additionally, 1,194 controls matching in self-reported ethnic background were collected. All individuals were genotyped with the HumanHap500v1 array [45, 46].

For the rheumatoid arthritis study, we utilized gender as environmental covariate. In the lung cancer study, age and smoking exposure, measured in pack years, were also considered. To determine the pack year, one multiplies the number of packs of cigarettes smoked per day by the number of years an individual has smoked.

All GWAS data were subjected to strict quality control. Only individuals with a genotype call rate of at least 95% were considered. SNPs with more than 10% missing values or with a minor allele frequency (MAF) below 0.1% were excluded from further analysis. Missing values in remaining markers were imputed with BEAGLE [47]. No SNPs beyond the original chip were imputed. The base pair positions of all SNPs were updated to NCBI build 38 using the Ensembl database [48], which was accessed using the R package biomaRt [49, 50]. Gene start and end positions were extracted from the same database, also using NCBI build 38. SNPs with no unique position were excluded. Refer to Table 3 for an overview of the study characteristics. Note that, during analysis, only SNPs mapped to genes within pathways were considered. The assignment of SNPs to genes was based on their base pair location and gene boundaries. SNPs closely located to each other are often in linkage disequilibrium (LD). For SNP annotation, we specified gene regions including LD-blocks extending beyond gene boundaries, as recommended in [51].

The KEGG database groups pathways in disjoint subsets according to their biological functionality. In the analysis of the rheumatoid arthritis and lung cancer data, we used a subgroup of 73 pathways connected to human diseases (see Table 4). The information on this group of pathways was downloaded in April 2016. An offset model containing only the environmental covariates was fitted for each of the studies to serve as start model for the kernel boosting of pathways.

For each pathway analyzed, the network-based kernel function with 4 degrees of freedom served as base-learner. The optimal number of iterations *m*_stop_ was derived via 20-fold subsampling and the default step length of 0.1 was used. For the purpose of comparison, each of the pathways considered in GWAS data analysis was also tested individually on the corresponding data using the LKMT. The same environmental variables that were used in the offset model for boosting were also considered for the LKMT. Prediction accuracy was measured by the misclassification rate and the area under the ROC curve (AUC) for both datasets. Of note, prediction accuracy is influenced by the applied model but also by the dataset at hand, that is, the amount of information contained in the data. Additionally, we provided the cross-validation results, that is, the (average) negative binomial likelihood on the data that was not used for model fitting (see Supplementary Material 1, Section B, for these results).

## 4. Results

### 4.1. Simulation Results

We compared the number of pathways each approach identified as associated with disease risk and considered the respective overlap in the results. The noninformative genetic data simulation comprised genotype data for 50 pathways and 1,000 individuals. Figure 3 displays the percentage of runs in which a pathway was selected. We can observe that the application of kernel boosting to these data does not lead to a high selection frequency for any pathway. Selection of pathways appears to be distributed randomly across all networks, not suggesting any clearly recognizable association with disease status. Note that, in kernel boosting, we do not conduct tests to evaluate the pathways' influence but select pathways based on their predictive performance. Thus, we cannot calculate a type I error to evaluate our method's performance. However, we can quantify the empirical type I error. Within 100 simulation runs on 50 pathways, a total number of 88 false selections occurred. Thus, a pathway was falsely selected in 1.76% of all possible cases. In 51 out of the 100 simulation runs, no single pathway was chosen by the algorithm. Hence, we conclude that kernel boosting can be trusted to reliably avoid false positive selections in noninformative data.

**Figure 4:**
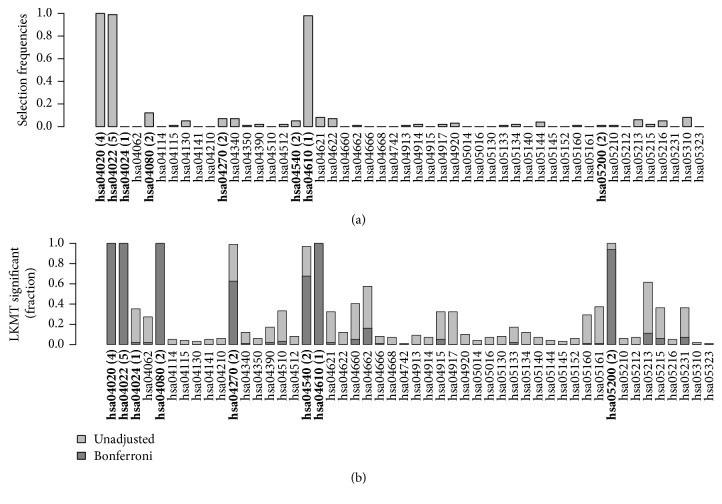
Relative frequency of datasets in which a pathway was selected using (a) kernel boosting (*n* = 2000, RR = 1.5) and (b) LKMT (*n* = 2000, RR = 1.5) for a sample size of 2000 individuals. Pathways including effect genes are labeled in bold; numbers in brackets denote the count of included influential genes within the pathway. All effects were simulated with a relative risk of 1.5 per allele.

**Figure 5:**
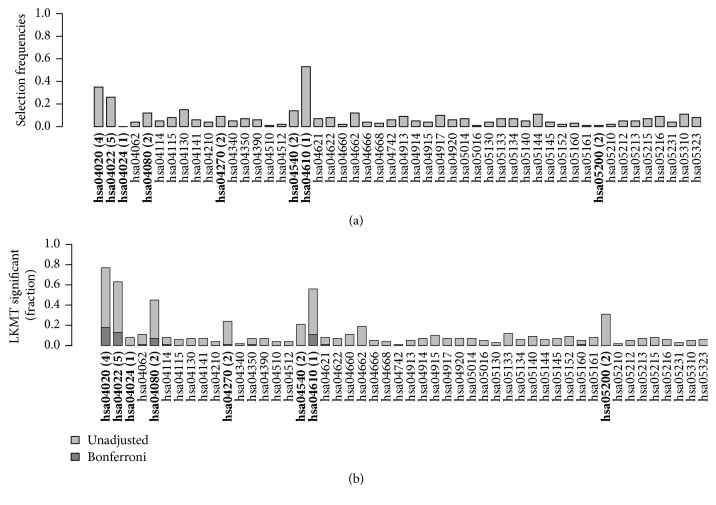
Relative frequency of datasets in which a pathway was selected using (a) kernel boosting (*n* = 500, RR = 1.5) and (b) LKMT (*n* = 500, RR = 1.5) for a sample size of 500 individuals. Pathways including effect genes are labeled in bold; numbers in brackets denote the count of included influential genes within the pathway. All effects were simulated with a relative risk of 1.5 per allele.

Figures 4 and 5 compare the results of effect simulations with a relative risk of 1.5 per allele for 1,000 cases and 1,000 controls to those for 250 cases and 250 controls. (a) in each figure contains barplots indicating selection frequencies of the 50 pathways across all simulation runs when applying kernel boosting to the corresponding simulation scenario. (b) compares these results with the selection frequencies using the LKMT. Here, both the percentages of results with a *p* value below 0.05 (lighter grey bars) and those with *p* values below the Bonferroni-corrected significance level of 0.001 (darker grey bars) are indicated. Pathways containing influential genes are additionally highlighted in italics.

The results of kernel boosting in the sample of 2,000 individuals (Figure 4(a)) display three pathways clearly identified as influential on the clinical outcome, as their selection frequency is close to 100%. These are the pathways originally chosen to include genetic effects,* hsa04020* and* hsa04022*, and the pathway* hsa04610*. It seems that the latter pathway is able to depict some of the information of the influential gene more effectively than the causal pathway for which it was originally simulated. This can be explained, as hsa04610 has the highest transitivity (0.14), also known as global clustering coefficient, of all simulation pathways and contains an effect gene. As the network kernel was designed to work especially well in detection of interconnected genetic effects, the causal gene is identified very well in the pathway when using this base-learner. Note that the same pathway did not stand out in the noninformative simulation scenario. Thus, we conclude that high transitivity facilitates the detection of causal effects when using the network-based kernel but does not lead to false positives (i.e., here, pathways which do not contain any effect gene). Several other pathways were also selected, but only with very low frequencies. In the same simulation scenario, the LKMT had very high power to detect the two pathways simulated to affect disease risk, however, also detected other pathways including any of the causal genes on the Bonferroni-adjusted significance level (Figure 4(b)). Three of the six other effect-containing pathways were selected in almost 100% of the replications and two of the remaining ones in more than 60% and one other pathway which contained an effect gene was hardly selected.

Overall, this indicates that kernel boosting can identify the pathways with the most explanatory power with respect to disease status and is less likely than LKMT to select pathways due to overlapping effect genes (see [6] for a discussion). The reason can be found in the multivariate nature of the kernel boosting approach, in which pathways are not tested separately for their influence, but a multivariate model is fitted to incorporate multiple influential predictors at the same time.

Figure 5(a) reveals that the selection frequencies of associated pathways drop noticeably when sample size decreases. The same three pathways as in the larger sample reached the highest selection frequencies but here only between 20% and 60%. Simultaneously, the number of selections across nonassociated pathways increased slightly compared to the larger sample. This indicates that a reduction in sample size leads to less clear identification of the main influential pathways by kernel boosting. In Figure 5(b), we notice a similar behaviour of the selection frequency in LKMT analysis. Here again, the power to identify pathways, previously well detected in the larger sample, drops clearly with the smaller dataset. Regarding the percentage of detected pathways on the Bonferroni-corrected significance level, the drop is even more pronounced in the LKMT than for kernel boosting. This indicates that kernel boosting is less strongly influenced by sample size and may have greater potential in the identification of causal effects in smaller datasets for which the LKMT is underpowered.

**Figure 6:**
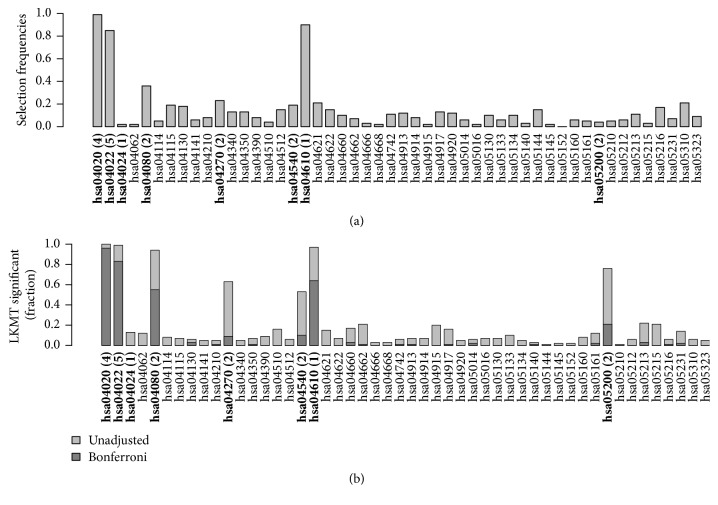
Relative frequency of datasets in which a pathway was selected using (a) kernel boosting (*n* = 1000, RR = 1.5) and (b) LKMT (*n* = 1000, RR = 1.5) for sample sizes of 1000 individuals. Effect strength was set to relative risks of 1.5 per allele. Pathways including effect genes are labeled in bold; numbers in brackets denote the count of included influential genes within the pathway.

**Figure 7:**
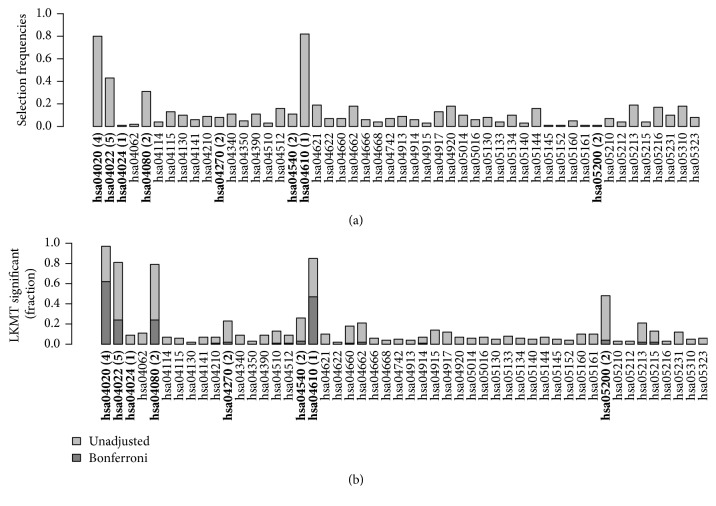
Relative frequency of datasets in which a pathway was selected using (a) kernel boosting (*n* = 1000, RR = 1.1) and (b) LKMT (*n* = 1000, RR = 1.1) for sample sizes of 1000 individuals. Effect strength was set to relative risks of 1.1 per allele. Pathways including effect genes are labeled in bold; numbers in brackets denote the count of included influential genes within the pathway.

Figures 6 and 7 compare the results of kernel boosting and the LKMT for differing effect sizes in equally sized samples of 1,000 individuals. The graphics are structured as Figures 4 and 5, with kernel boosting selection frequencies plotted in (a) and LKMT selection frequencies in (b).  Figure 6 contains a simulation scenario with relative risk of 1.5 per causal allele and Figure 7 the results for a relative risk of 1.1 per allele. Again, pathways containing influential genes are additionally highlighted.

In the kernel boosting plot in Figure 6(a), the three pathways standing out in Figure 4 again reached very high selection frequencies. All three bars decreased slightly in size compared to the scenario with 2,000 individuals but still illustrate selections in more than 80% of simulation runs. Selection frequencies of the other effect pathways increased compared to the scenarios in Figure 4. However, as selections across noninfluential pathways occurred more frequently here, they cannot clearly be identified as influential based on their selection frequencies alone. In the LKMT analysis of this sample, the power to detect causal effects noticeably drops compared to the 2,000 individuals' sample illustrated in Figure 4(b). Comparing Figures 6with 7, we can see a drop in selection frequencies as well as in power to detect associated pathways. In Figure 6, the two chosen effect pathways were detected in almost 100% and around 80% of simulation runs for both methods. In Figure 7, we observe that kernel boosting reaches selection frequencies of around 80% and 40%, while the LKMT with Bonferroni correction only achieves selection frequencies slightly greater than 60% and 20%, respectively. In a similar fashion to the results of the scenarios compared in Figures 4 and 5, both methods have higher power to detect associations for stronger effects; however the drop in power is less pronounced for kernel boosting. We conclude that kernel boosting firstly has no inferior performance in terms of power compared to the LKMT. It may even prove more likely to identify influential pathways with smaller genetic effects as it overcomes the multiple testing problem. Secondly, we infer that, in contrast to single-pathway testing approaches, kernel boosting has the ability to discriminate crucial biological processes associated with disease risk from effects included in pathways only due to overlapping genes.

### 4.2. GWAS Analysis Results

Kernel boosting on the human disease pathways in the lung cancer dataset resulted in selection of only the prion diseases pathway (KEGG id hsa05020). No other pathway was selected. The misclassification error of the tuned boosting model for lung cancer (evaluated at the optimal cut point as defined by the minimal Youden index) was 24.5% and the AUC was 0.785. The ROC curve and the cross-validation results are presented in the Supplementary Material 1, Section B. The LKMT with network-based kernel did not detect any associated pathway on the Bonferroni-corrected significance level. The prion diseases pathway appears ranked 20 out of 73 pathways, when sorting pathways according to ascending Bonferroni-corrected *p* values. Prions are misfolded proteins capable of changing the structure of other, properly folded proteins into their own incorrect prion structure. They have mostly been reported in connection with neurodegenerative diseases [52]. Nevertheless, a connection with different forms of cancer has also previously been suspected [53, 54]. A full table of results from the analysis of the lung cancer dataset can be found in Supplementary Material 1, Section B.

As expected, analysis of the rheumatoid arthritis dataset discovered a variety of pathways (compare results in [13]). Kernel boosting constructed an explanatory model for disease status based on 32 selected pathways (see pathways written in italics in Table 4). It is well known that genes belonging to the human leukocyte antigen (HLA) complex are highly correlated with rheumatoid arthritis [55]. The HLA family, located on the short arm of chromosome 6, is a highly polymorphic genetic system mainly responsible for the regulation of the immune system [56]. In the human disease class, 18 pathways contain at least one of the HLA genes. These pathways are marked with an asterisk in Table 4. Between the 18 pathways containing HLA genes and the 32 pathways selected by kernel boosting, there is an overlap of 10 pathways. This may be explained by the multivariate nature of the method, in which only the pathway most clearly representing a particular genetic effect will be selected, conditionally on previously selected effects. Testing the human disease pathways' influence on disease status with the LKMT resulted in a large number of 46 significantly associated pathways out of 73 pathways after Bonferroni correction (see pathways with *p* values in Table 4). These included almost all HLA pathways (15 out of 18). The more specific identification of influential pathways by kernel boosting provides a more complete basis to the understanding of the crucial biological processes involved in disease susceptibility. The misclassification error of the tuned boosting model for rheumatoid arthritis (evaluated at the optimal cut point as defined by the minimal Youden index) was 22.7% and the AUC was 0.850. The ROC curve and the cross-validation results are presented in Supplementary Material 1, Section B.

## 5. Discussion

We extend a successful method for single-pathway tests to a multivariate selection approach for simultaneous analysis of several pathways. The resulting kernel boosting method benefits from the advantages of a kernel-based analysis, while at the same time overcomes some of the limitations inherent to testing procedures.

Moreover, our multivariable approach to GWAS data analysis does not provide *p* values, which only provide limited information on the relevance of a genetic effect. A more meaningful result would be an effect measure for the investigated trait or better still the ability to predict an outcome. Kernel boosting facilitates prediction, based on the selected influential variables, as was elucidated in the application where the overall prediction accuracy of each of the models was reported. Thus, it is also possible to interpret the influence of a specific genetic alteration by comparing the change in the predicted outcomes. A high degree of prediction accuracy for the model is ensured through the convenient evaluation of its performance on subsamples of the investigated dataset. This procedure usually results in good prediction accuracy and a sparse model.

Owing to the built-in shrinkage, our boosting approach is capable of dealing with correlated effects. Hence, correlated pathways, which partly include the same genes, can be handled within this framework. Thanks to the multivariable nature of the approach, only the best-fitting pathways, evaluated in terms of prediction accuracy, will be chosen to enter the model. Thus, only the pathway most clearly representing a particular genetic effect will be selected, depending on those pathways selected previously. Our observations support the statement by de Leeuw et al. [57] that competitive gene-set analysis methods (multivariate approach, pathways in competition), in contrast to self-contained approaches (univariate approach, one pathway at a time), can potentially differentiate widely spread heritability of polygenetic outcomes from causal biological processes. This property can be very helpful in the identification and understanding of specific biological functions involved in disease susceptibility.

We consider pathways as analysis units; however various other options exist. Single SNPs in transcribed or untranscribed regions, and SNP sets aggregated to represent a specific genomic region, environmental variables, or other variables, may be investigated and even combined arbitrarily within one model. For example, the application of our method to the genes comprising a pathway may help to identify key influential genes within the network (for gene boosting, see also the work of Ma et al. [58]; for good overviews of feature selection methods and machine learning tools in bioinformatics refer to [59, 60]). Known influential factors may be embedded in an initial model prior to the selection procedure to adjust for environmental or genetic effects. Furthermore, the considered effects can be incorporated into the model via a multitude of possible base-learners.

The choice of a base-learner can influence effect selections. We observed this behaviour during the simulations, in which the highly connected pathway containing only one effect gene was identified owing to the network-based kernel's high power on interconnected effects. Thus, the well-considered selection of base-learners to be utilized is advisable. We account for the high complexity of possible gene interactions in pathways via the use of a kernel function, which accounts for additive and interaction effects. Such a kernel function will likely lead to a higher degree of prediction accuracy than a simple linear kernel. The application of our method to GWAS datasets on rheumatoid arthritis and lung cancer returned biologically plausible results. Particularly with the rheumatoid arthritis dataset, the number of identified pathways could be reduced considerably compared to single-pathway tests. While the LKMT resulted in 46 significantly associated pathways, kernel boosting narrowed the selection down to 32 pathways. Genes within the HLA region are known to have a strong influence on rheumatoid arthritis. Their effects can reach far across pathways, such that the LKMT detects many pathways including HLA genes as significantly associated. Boosting seems to help to pinpoint down signals even among those pathways and reduces the number of identified pathways to a more reasonable level.

Our results indicate that kernel boosting outperforms single kernel machine tests, as exemplified by the LKMT, in certain genetic scenarios. It may help to discriminate causal biological processes from isolated effects included in pathways only due to gene overlap and facilitate discovering weak signals, especially in studies of limited size. This is of particular interest in the investigation of rare diseases and disease subtypes, in which established methods often fail to find any significantly associated pathways owing to a lack of power.

Datasets of the size investigated here can be analyzed with kernel boosting quite efficiently on current high-performance cluster computing (HPCC) systems. However, such analysis of very large datasets places a rather high demand even on the most powerful HPCC systems to date. Usually, our kernel base-learners are based on the pairwise similarities of all observations. This leads to *n* × *n* similarity matrices as design matrices and hence to parameter vectors **γ** of size *n*. Instead of using all pairwise similarities, it is possible to compute the similarities only to a representative subset of the observations, or so-called knots. These knots can be chosen as subset of the observations which covers the complete observation space (space-filling algorithm; see [33, 61, 62]). Consequently, we obtain reduced-rank design matrices of dimension $n\times \tilde{n}$, where $\tilde{n}$ is the number of knots, and a parameter vector of size $\tilde{n}$. This reduces the computational burden for the construction of the kernel base-learners and effect estimation and makes kernel-based methods even feasible in situations with many observations. The exact number of observations that can be processed depends, among others, on the considered number of individuals, SNPs, base-learners chosen, and the available hardware.

Kernel boosting constitutes a new and potentially powerful tool in the analysis of GWAS data. It offers a highly flexible and extensible framework, suitable for a wide range of application scenarios. We account for the high complexity of possible gene interactions via the use of kernel functions, while reducing the complexity of the resulting model with the built-in shrinkage of the boosting approach. The resulting model enables us to predict traits and returns more meaningful results than a testing procedure. We conclude that kernel boosting is a suitable methodological addition for the analysis of GWAS, which supports the detection and interpretation of genetic risk factors influencing disease susceptibility.

## Supplementary Material

Supplement 1 (supplemental text, PDF): the supplemental text gives further details on the conducted analysis. It contains additional information on the simulation study including explanations on the choice of the maximum number of iterations, an in-depth analysis of the selection of relevant pathways, details on the computational requirements, and details on the effect pathways used for the simulation study. The supplement also contains further results of the data analyses on lung cancer and rheumatoid arthritis.
Supplement 2 (code and data, ZIP-archive): the supplemental code and data files provide an exemplary application of the kernel boosting method to a simulated data set. A readme file and comments in the code highlight all important aspects and explain the analysis steps which are required.

## Figures and Tables

**Table 1:** Description of network properties for pathway topology of pathways used in simulations, compared to the properties of the two effect pathways hsa04020 and hsa04022. Nodes equal the number of included genes, links give the number of interactions, inhibition links the count of interactions of inhibiting type, the average degree of a node is the mean number of adjacent edges, density is the ratio between numbers of existing links and possible links, diameter denotes the distance to the farthest node in the graph, transitivity (also called cluster coefficient) calculates the probability of adjacent vertices of a vertex being connected, and signed transitivity considers the type of interaction in this calculation.

	Min	Mean	Median	Max	hsa04020	hsa04022
Nodes	29.00	103.60	86.5	398.00	180.00	167.00
Links	1.00	197.81	87.5	1493.00	297.00	372.00
Inhibition links	0.00	27.08	10.50	148.00	7.00	67.00
Average degree	0.07	3.18	2.36	15.62	3.30	4.46
Density	0.00	0.03	0.03	0.16	0.02	0.03
Inhibition degree	0.00	0.52	0.24	2.62	0.08	0.80
Diameter	1.00	7.36	7.00	18.00	6.00	7.00
Transitivity	0.00	0.02	0.00	0.14	0.00	0.03
Signed transitivity	−0.02	0.01	0.00	0.10	0.00	0.03

**Table 2:** Counts of included influential genes within pathways used for simulation purposes. Pathways without simulated causal genes are not displayed.

KEGG id	Name of pathway	Effect genes included
hsa04020	Calcium signaling pathway	4
hsa04022	cGMP-PKG signaling pathway	5
hsa04024	cAMP signaling pathway	1
hsa04080	Neuroactive ligand-receptor interaction	2
hsa04270	Vascular smooth muscle contraction	2
hsa04540	Gap junction	2
hsa04610	Complement and coagulation cascades	1
hsa05200	Pathways in cancer	2

**Table 3:** Characteristics of analyzed GWAS datasets. Numbers of case and control individuals after quality control and SNP numbers for several analysis stages are displayed. Preprocessing of SNPs included quality control of genotype data, as well as updating genomic SNP positions according to the latest information (genomic build 38). The last column indicates the total number of all SNPs annotated to a pathway under investigation.

Study	Cases/controls	SNPs genotyped	SNPs after preprocessing	SNPs in analysis
Lung cancer	467/468	561,466	533,062	148,938
Rheumatoid arthritis	866/1189	545,080	491,695	137,839

**Table 4:** KEGG pathways in the human diseases class as downloaded in April 2016. Pathways are sorted according to *p* value, derived from LKMT application on the rheumatoid arthritis dataset, in ascending order. *p* values for pathways significantly associated after Bonferroni correction are listed. Pathways selected by kernel boosting on the same dataset are marked in italics. Pathways containing one or several genes belonging to the HLA complex are marked with an asterisk behind the id number.

KEGG id	Name of pathway	*p* value
hsa05133	Pertussis	1.562 × 10^−32^
*hsa05150* ^*∗*^	*Staphylococcus aureus infection *	1.029 × 10^−30^
hsa04933	AGE-RAGE signaling pathway in diabetic complications	3.877 × 10^−17^
*hsa05169* ^*∗*^	* Epstein-Barr virus infection *	2.651 × 10^−16^
*hsa05144 *	* Malaria *	3.087 × 10^−15^
*hsa05206 *	* MicroRNAs in cancer *	3.969 × 10^−15^
*hsa05330* ^*∗*^	* Allograft rejection *	4.131 × 10^−12^
*hsa05200 *	* Pathways in cancer *	7.695 × 10^−11^
*hsa05166* ^*∗*^	* HTLV-I infection *	1.344 × 10^−11^
hsa05030	Cocaine addiction	1.353 × 10^−11^
*hsa05323* ^*∗*^	* Rheumatoid arthritis *	1.466 × 10^−11^
hsa05310^*∗*^	Asthma	2.268 × 10^−11^
hsa05134	Legionellosis	1.699 × 10^−05^
*hsa04940* ^*∗*^	* Type I diabetes mellitus *	3.591 × 10^−10^
hsa05031	Amphetamine addiction	3.735 × 10^−10^
hsa05145^*∗*^	Toxoplasmosis	4.555 × 10^−10^
*hsa05203* ^*∗*^	*Viral carcinogenesis *	1.814 × 10^−09^
hsa05332^*∗*^	Graft-versus-host disease	5.940 × 10^−09^
*hsa05020 *	*Prion diseases *	1.530 × 10^−07^
hsa05143	African trypanosomiasis	2.114 × 10^−07^
hsa05222	Small-cell lung cancer	3.782 × 10^−07^
hsa05205	Proteoglycans in cancer	1.236 × 10^−06^
*hsa05322* ^*∗*^	*Systemic lupus erythematosus *	1.702 × 10^−06^
*hsa05161 *	*Hepatitis B *	1.757 × 10^−06^
*hsa05410 *	*Hypertrophic cardiomyopathy (HCM) *	1.980 × 10^−06^
hsa05010	Alzheimer's disease	7.234 × 10^−06^
hsa05142	Chagas disease (American trypanosomiasis)	1.048 × 10^−05^
*hsa05168* ^*∗*^	*Herpes simplex infection *	1.109 × 10^−05^
*hsa05012 *	*Parkinson's disease *	1.368 × 10^−05^
hsa04932	Nonalcoholic fatty liver disease (NAFLD)	1.823 × 10^−05^
hsa05321^*∗*^	Inflammatory bowel disease (IBD)	2.124 × 10^−05^
*hsa04931 *	*Insulin resistance *	3.625 × 10^−05^
*hsa05219 *	*Bladder cancer *	4.133 × 10^−05^
*hsa05215 *	*Prostate cancer *	4.220 × 10^−05^
hsa05202	Transcriptional misregulation in cancer	7.697 × 10^−05^
hsa05220	Chronic myeloid leukemia	8.464 × 10^−05^
hsa05146	Amoebiasis	1.003 × 10^−04^
hsa05414	Dilated cardiomyopathy	1.014 × 10^−04^
hsa05231	Choline metabolism in cancer	1.504 × 10^−04^
*hsa05032 *	*Morphine addiction *	1.672 × 10^−04^
*hsa05162 *	*Measles *	2.390 × 10^−04^
hsa05214	Glioma	2.506 × 10^−04^
hsa05164^*∗*^	Influenza A	2.720 × 10^−04^
*hsa05416* ^*∗*^	*Viral myocarditis *	3.384 × 10^−04^
*hsa05132 *	*Salmonella infection *	5.147 × 10^−04^
hsa05014	Amyotrophic lateral sclerosis (ALS)	5.568 × 10^−04^
hsa04930	Type II diabetes mellitus	Not significant
hsa05218	Melanoma	Not significant
hsa05140^*∗*^	Leishmaniasis	Not significant
*hsa05213 *	*Endometrial cancer *	Not significant
*hsa05211 *	*Renal cell carcinoma *	Not significant
*hsa05340 *	*Primary immunodeficiency *	Not significant
*hsa05160 *	*Hepatitis C *	Not significant
hsa05212	Pancreatic cancer	Not significant
hsa05016	Huntington's disease	Not significant
hsa05221	Acute myeloid leukemia	Not significant
*hsa04950 *	*Maturity onset diabetes of the young *	Not significant
*hsa05412 *	*Arrhythmogenic right ventricular cardiomyopathy (ARVC) *	Not significant
hsa05223	Non-small-cell lung cancer	Not significant
hsa05034	Alcoholism	Not significant
hsa05130	Pathogenic Escherichia coli infection	Not significant
hsa05120	Epithelial cell signaling in Helicobacter pylori infection	Not significant
*hsa05131 *	*Shigellosis *	Not significant
*hsa05204 *	*Chemical carcinogenesis *	Not significant
hsa05100	Bacterial invasion of epithelial cells	Not significant
hsa05216	Thyroid cancer	Not significant
hsa05152^*∗*^	Tuberculosis	Not significant
hsa05210	Colorectal cancer	Not significant
hsa05230	Central carbon metabolism in cancer	Not significant
*hsa05217 *	*Basal cell carcinoma *	Not significant
hsa05320^*∗*^	Autoimmune thyroid disease	Not significant
hsa05033	Nicotine addiction	Not significant
hsa05110	Vibrio cholerae infection	Not significant
