# Supplementary material for: Pathway-Based Kernel Boosting for the Analysis of Genome-Wide Association Studies
Source: Comput Math Methods Med. 2017 Jul 13;2017:6742763. doi: 10.1155/2017/6742763 (PMC5530424; doi:10.1155/2017/6742763)
Supplement: Supplementary file 1 — Supplement 1 (supplemental text, PDF): the supplemental text gives further details on the conducted analysis. It contains additional information on the simulation study including explanations on the choice of the maximum number of iterations, an in-depth analysis of the selection of relevant pathways, details on the computational requirements, and details on the effect pathways used for the simulation study. The supplement also contains further results of the data analyses on lung cancer and rheumatoid arthritis. Supplement 2 (code and data, ZIP-archive): the supplemental code and data files provide an exemplary application of the kernel boosting method to a simulated data set. A readme file and comments in the code highlight all important aspects and explain the analysis steps which are required. [file 6742763.f1.pdf]

Supplemental Material 1

for

Pathway-Based Kernel Boosting for the Analysis of Genome-Wide Association Studies

Stefanie Friedrichs[1,*], Juliane Manitz[2,3], Patricia Burger[1], Christopher I. Amos[4], Angela Risch[5,6,7], Jenny Chang-Claude[8], H.-Erich Wichmann[9,10,11], Thomas Kneib[2], Heike Bickeböller[1], and Benjamin Hofner[12, 13]

[1]Institute of Genetic Epidemiology, University Medical Centre, Georg-August University Göttingen, Göttingen, Germany.
[2]Department of Statistics and Econometrics, Georg-August University Göttingen, Göttingen, Germany
[3]Department of Mathematics and Statistics Boston University, Bosten, USA
[4]Department of Community and Family Medicine, Geisel School of Medicine, Dartmouth College, Lebanon, NH, United States of America
[5]Division of Molecular Biology, University of Salzburg, Austria
[6]Translational Lung Research Center Heidelberg (TLRC-H), Member of the German Center for Lung Research (DZL), Heidelberg, Germany
[7]Division of Epigenomics and Cancer Risk Factors, DKFZ German Cancer Research Center, Heidelberg, Germany
[8]Division of Cancer Epidemiology, German Cancer Research Center (DKFZ), Heidelberg, Germany
[9]Institute of Medical Informatics, Biometry and Epidemiology, Chair of Epidemiology, Ludwig-Maximilians University, Munich, Germany
[10]Helmholtz Center Munich, Institute of Epidemiology 2, Germany
[11]Institute of Medical Statistics and Epidemiology, Technical University Munich, Germany
[12]Department of Medical Informatics, Biometry and Epidemiology, Friedrich-Alexander-Universität Erlangen-Nürnberg, Erlangen, Germany
[13]Section Biostatistics, Paul-Ehrlich-Institut, Langen, Germany

A Additional Analysis of Simulation Study

A.1 Choice and distribution of m_{stop}

In the primary analysis of the simulation study, we tried to convey a clear picture of the selection properties of the boosting algorithm, which can be easily related to the selection of pathways based on LKMT tests. As such we chose a relatively small number of boosting iterations to check if the influential pathways are selected early on and if they can be clearly distinguished from non-influential pathways. Hence, in the analysis of simulation results reported in the manuscript, the ideal number of iterations m_{stop} was determined within a search range of 0 to 200. Specifying a (relatively small) maximum number of possible iterations might force an early stopping of the algorithm in some simulation runs.

To investigate this issue, we re-analysed all simulation scenarios with a larger number of maximal iterations permitted, in order to allow the algorithm to reach the optimal boosting iteration, i.e., to find an iteration m_{stop} such that the out-of-bag risk is minimal. The number of iterations needed usually depends on the strength of the signal (effect size), the number of informative base-learners and the number of observations. In our simulation study, the number of iterations was mainly influenced by the number of observations (but also, though to a lesser extend) by the effect size. For simulation scenarios up to 1000 individuals, we considered a maximum of 500 iterations, while for samples of 2000 individuals, the algorithm was allowed to perform up to 1000 iterations.

In Figure 1 we display the observed number of iterations required for each simulation scenario to reach the optimal prediction accuracy as measured by the cross-validated out-of-bag Binomial log-likelihood.

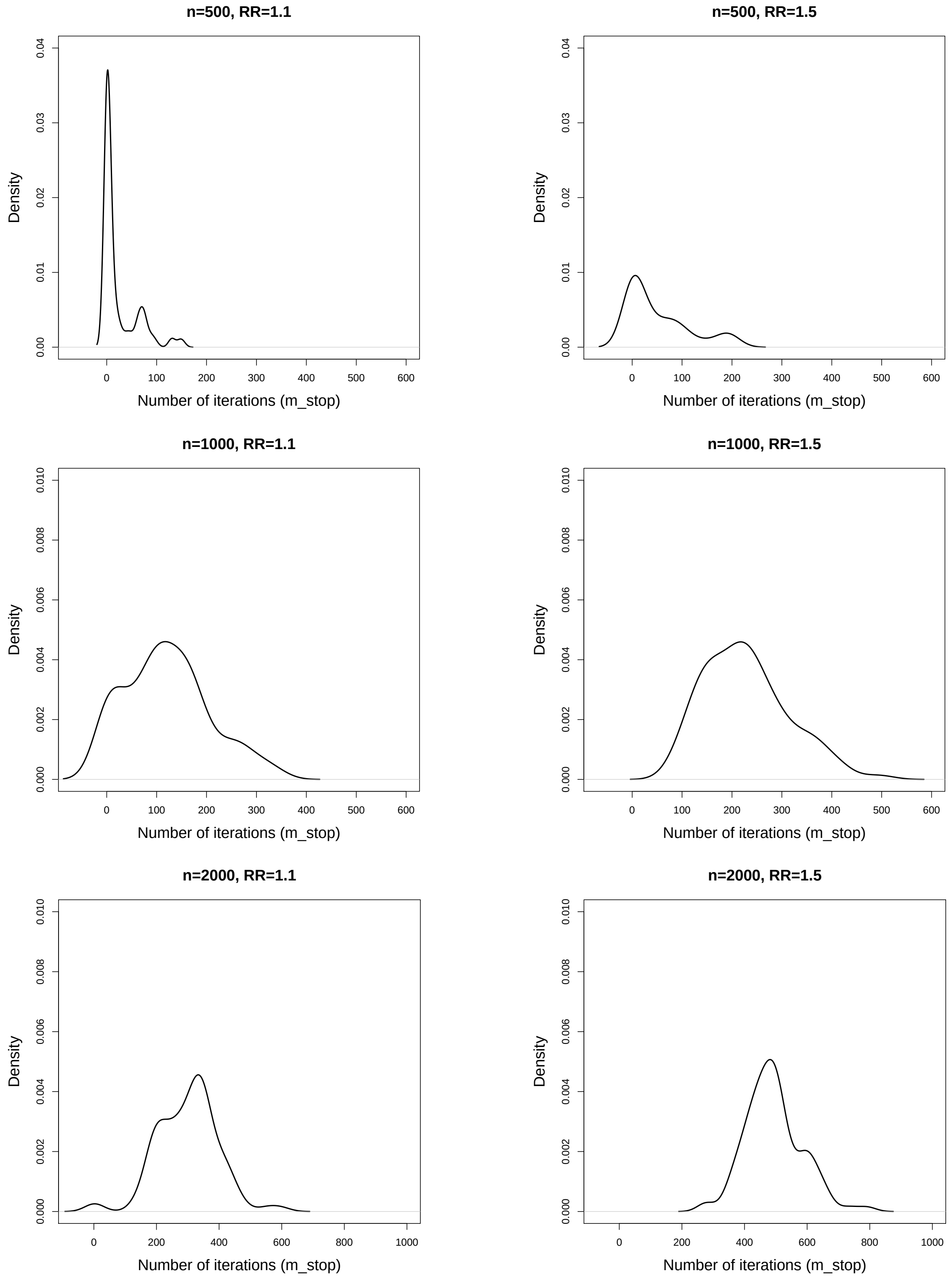

Figure 1: Kernel density estimates of the number of iterations (m_{stop}) in the 100 simulation runs for the different simulation scenarios.

A.2 Selection of Pathways

Increasing of the number of iterations, as discussed in the previous section, leads to an increase in runtime and likely results in the selections of additional pathways. Even though boosting tends to have a slow overfitting behavior [1, 2], at a certain point, non-influential effects are selected as well. This is more pronounced for data sets with many observations compared to the number of base-learners (i.e., "$n > p$"). Especially in later boosting iterations, it might happen that non-informative pathways are selected. However, these pathways are usually selected infrequently and with a small effect on the predicted outcome. Pathways selected early and often will have much more influence on the prediction.

The additional selections of causal and also non-causal pathways results in a less clear discrimination of influential biological processes. This disadvantage can be compensated for, however, by evaluating the results of kernel boosting in more detail. As the boosting algorithm can not only select a pathway once, but will usually select the same effect variable multiple times, if it is highly influential on the outcome, we can interpret the selection frequency of each pathway for a single simulated data set. This is one means to take the clinical relevance into account. Alternatively, one could consider the effect size, i.e., the size of the coefficient for linear base-learners or the norm of the coefficient vector for pathway kernel base-learners.

In the following paragraph, we assess the selection properties of the boosting algorithm when run until convergence. The upper panels of Figures 2 to 7 depict the relative selection frequencies of each base-learner averaged over all 100 simulation runs per scenario. Here, we firstly count how often each pathway has been selected in a single simulation run. This number is then transformed into a proportion of selections by deviding it with the chosen m_{stop} in the corresponding run. Secondly, these proportions per pathway are averaged across all 100 simulation runs. In this way, we are taking into account the relative importance of that effect. For comparisons the lower panel in each of the figures shows the relative frequency of simulation runs in which a base-learner was selected at least once. The latter plots are equal in structure to those in the paper, they merely show results for larger values of m_{stop}.

We can see, that for the simulation scenarios of 500 and 1000 individuals, no remarkable change was detected when increasing the maximum number of iterations. Especially in the simulation scenarios with 500 individuals, hardly any difference between top and lower barplots is visible (Figures 2 and 3). In simulation scenarios of 1000 individuals, depicted in Figures 4 and 5, we can see that the influential biological processes, represent by the two simulated effect pathways, are more precisely distinguished from non-causal pathways when also taking into account relative selection frequencies. For the scenario with 2000 individuals (Figure 7) we can see that considering relative selection frequencies has more impact in larger samples. Here a clear difference between the upper and lower barplot is visible. When only considering if a pathway was ever selected (lower row), influential and non-influential pathways can less clearly be discriminated. Additional evaluation of the relative selection frequency (top row) gives a much clearer picture and facilitates identification of the causal pathways. Note, that the top barplot for the scenario with 2000 individuals and a relative risk of 1.5 per allele (Figure 7) looks similar to Figure 4 in the Paper, which evaluated selections only on the same data for a smaller number of iterations. This means, that we can identify the influential pathways in a dataset with a noticeably reduction in computation time using early stopping.

We conclude that he discrimination of biologically relevant processes from gene overlaps is possible by letting the algorithm run until the optimal m_{stop} when taking not only into account if a pathway was selected, but also considering the relative selection frequencies. Using this approach, causal pathways were even more precisely distinguished from non-causal pathways than in the case of evaluating only if a pathway was seletced at least once or not.

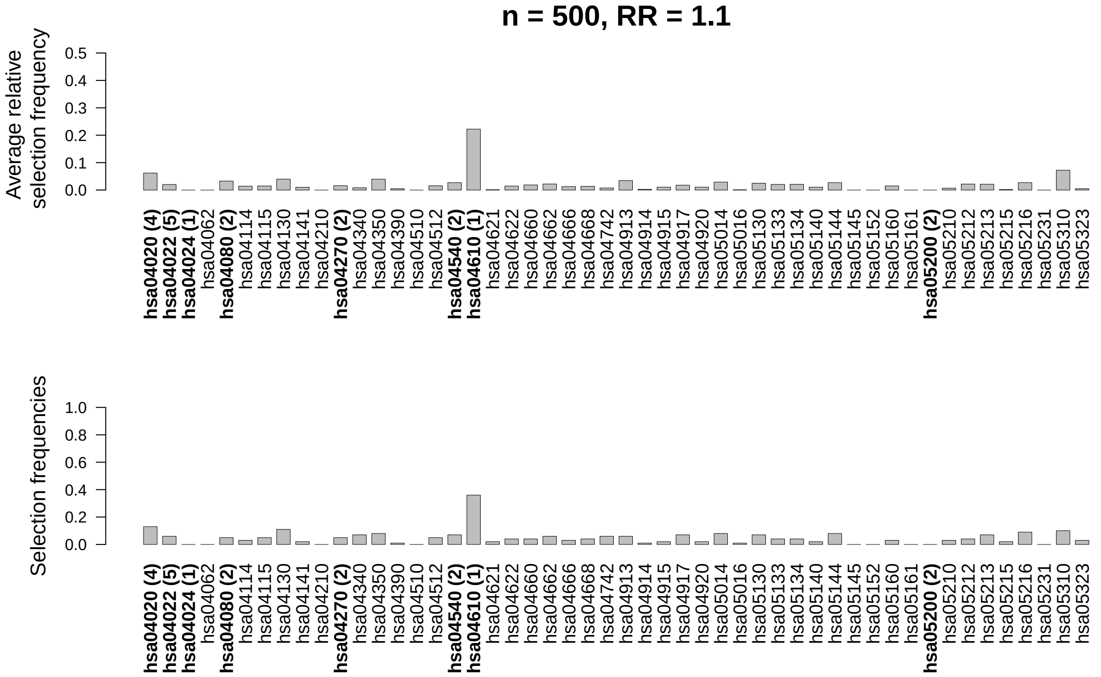

Figure 2: Barplots for the relative selection frequencies of each base-learner in a single run averaged over all 100 simulation runs (top) and relative frequencies of simulation runs in which a base-learner was selected at least once (bottom). The sample comprised 250 cases and 250 controls and the effect strength was set to relative risks of 1.1 per allele. Pathways including effect genes are labeled in bold; numbers in brackets denote the count of included influential genes within the pathway.

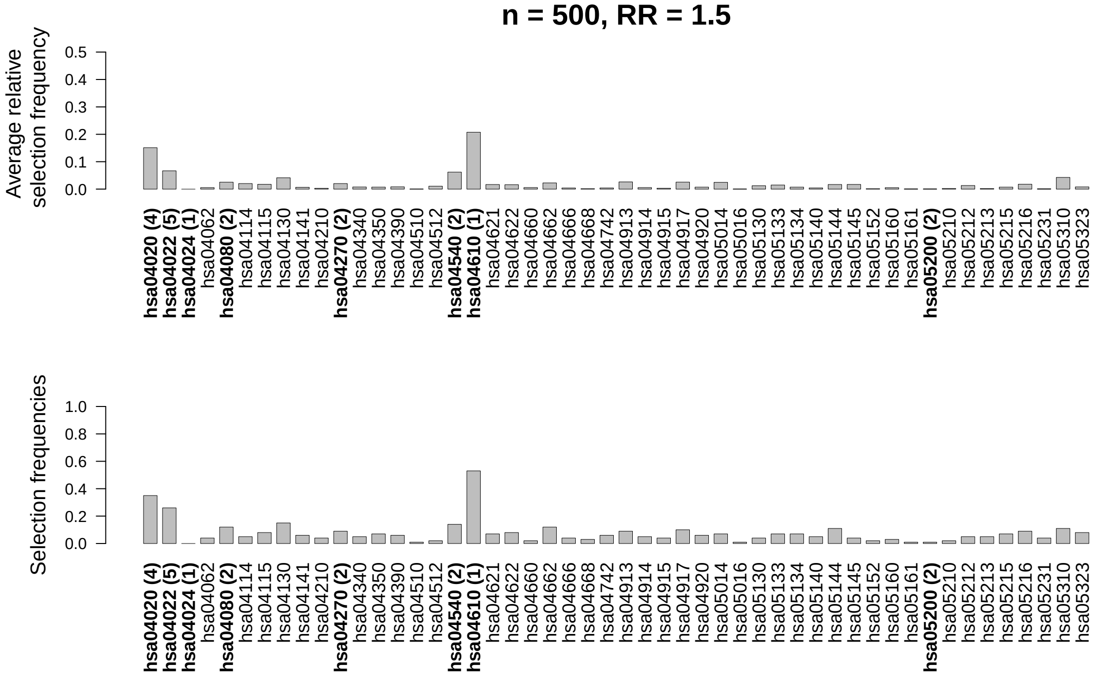

Figure 3: Barplots for the relative selection frequencies of each base-learner in a single run averaged over all 100 simulation runs (top) and relative frequencies of simulation runs in which a base-learner was selected at least once (bottom). The sample comprised 250 cases and 250 controls and the effect strength was set to relative risks of 1.5 per allele. Pathways including effect genes are labeled in bold; numbers in brackets denote the count of included influential genes within the pathway.

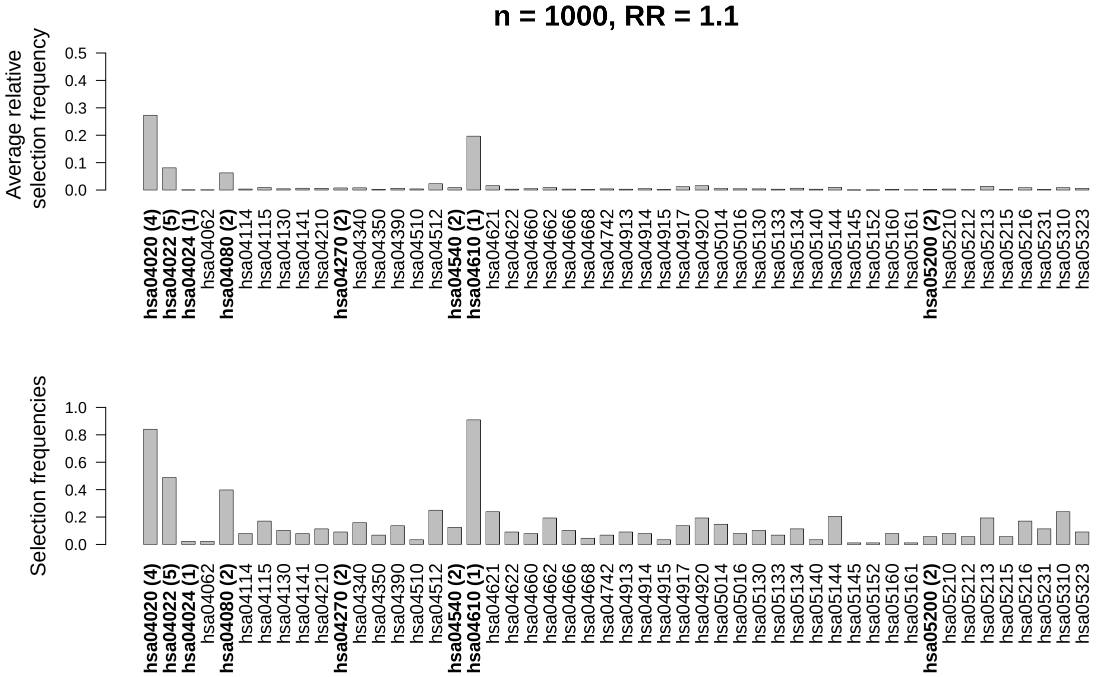

Figure 4: Barplots for the relative selection frequencies of each base-learner in a single run averaged over all 100 simulation runs (top) and relative frequencies of simulation runs in which a base-learner was selected at least once (bottom). The sample comprised 500 cases and 500 controls and the effect strength was set to relative risks of 1.1 per allele. Pathways including effect genes are labeled in bold; numbers in brackets denote the count of included influential genes within the pathway.

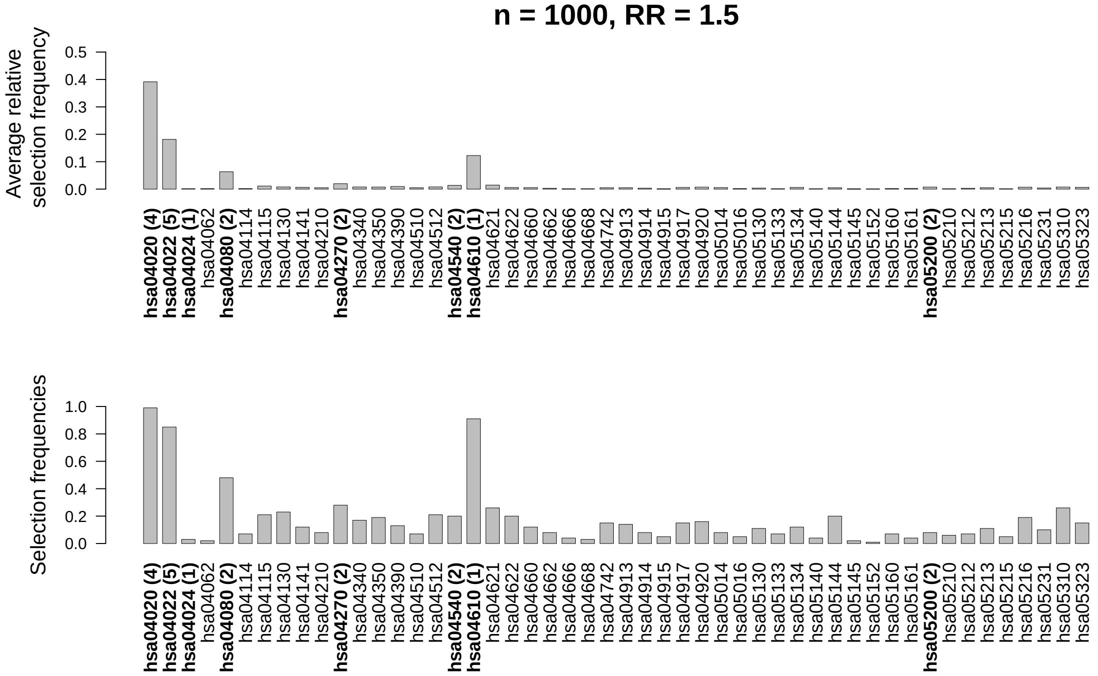

Figure 5: Barplots for the relative selection frequencies of each base-learner in a single run averaged over all 100 simulation runs (top) and relative frequencies of simulation runs in which a base-learner was selected at least once (bottom). The sample comprised 500 cases and 500 controls and the effect strength was set to relative risks of 1.5 per allele. Pathways including effect genes are labeled in bold; numbers in brackets denote the count of included influential genes within the pathway.

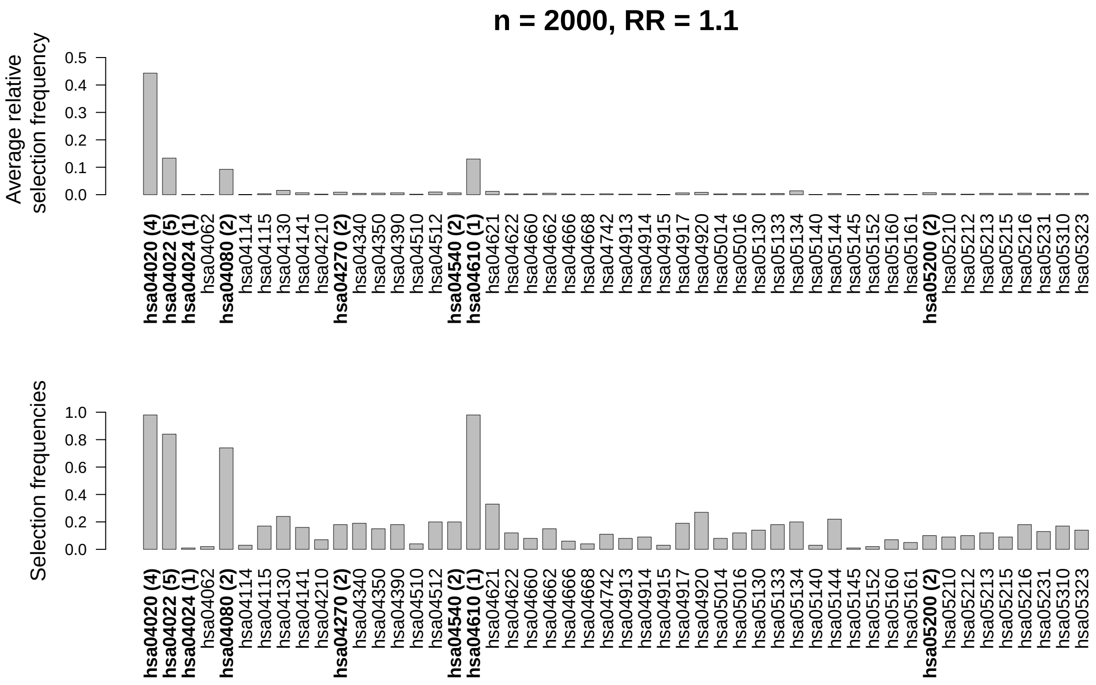

Figure 6: Barplots for the relative selection frequencies of each base-learner in a single run averaged over 100 simulation runs (top) and relative frequencies of simulation runs in which a base-learner was selected at least once (bottom). The sample comprised 1000 cases and 1000 controls and the effect strength was set to relative risks of 1.1 per allele. Pathways including effect genes are labeled in bold; numbers in brackets denote the count of included influential genes within the pathway.

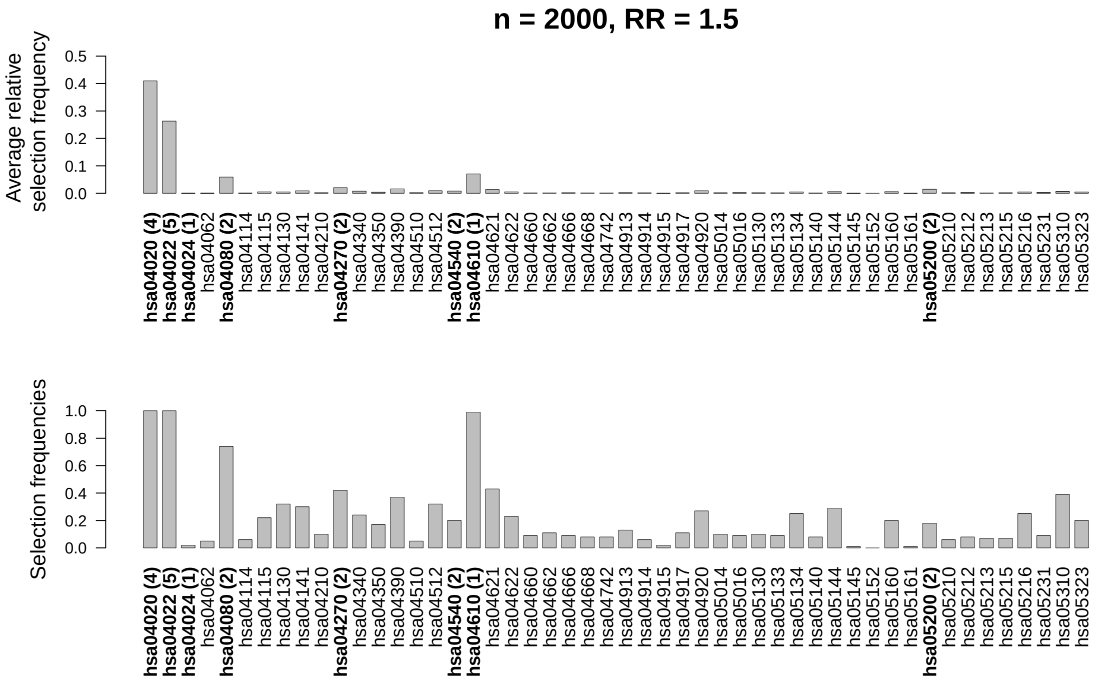

Figure 7: Barplots for the relative selection frequencies of each base-learner in a single run averaged over all 100 simulation runs (top) and relative frequencies of simulation runs in which a base-learner was selected at least once (bottom). The sample comprised 1000 cases and 1000 controls and the effect strength was set to relative risks of 1.5 per allele. Pathways including effect genes are labeled in bold; numbers in brackets denote the count of included influential genes within the pathway.

A.3 Computational Requirements

In the following, we provide run times and memory requirements for exemplary simulation runs. The measurements include the model fitting with 50 simulated pathways and 20-fold cross-validation to determine the optimal m_{stop}. Cross-validation was run in parallel on 20 cores. We report the runtime (time actually needed for the process), the CPU time (sum of run time over all CPUs used; approximates the runtime if the process was run sequentially) and maximum memory allocation:

- Kernel boosting for the simulation scenario with 500 individuals required a runtime of 12.8 minutes (corresponding CPU time 3.5 hours) as well as a maximum memory use of 11.6 GB to determine the optimal m_{stop} between 0 and 500.
- Analysis of the simulation scenario including 1000 individuals resulted in a runtime of 1.9 hours, equalling a CPU time of 24.9 hours, for the same search range of m_{stop}. The maximum memory use was approximately 40 GB.
- The simulation scenario with 2000 individuals needed a runtime of 23.3 hours (CPU time 340.6 hours), and utilized a maximum memory of 132 GB. Here, the ideal number of iterations was to be determined between 0 and 1000.

Note, that the actual runtime can vary (e.g. depending on the system, the CPU and the memory available). In practice, the runtime is significantly smaller than the CPU time, as can be seen above, as it is very easy to run the cross-validation in parallel. Of course, parallelization also requires a higher amount of memory. Hence, running the cross-validation sequentially will require less memory, but will take longer.

A.4 Details on Effect Pathways

A graphical display of the two networks that were simulated to contain effect genes is given in Figures 9 and 8.

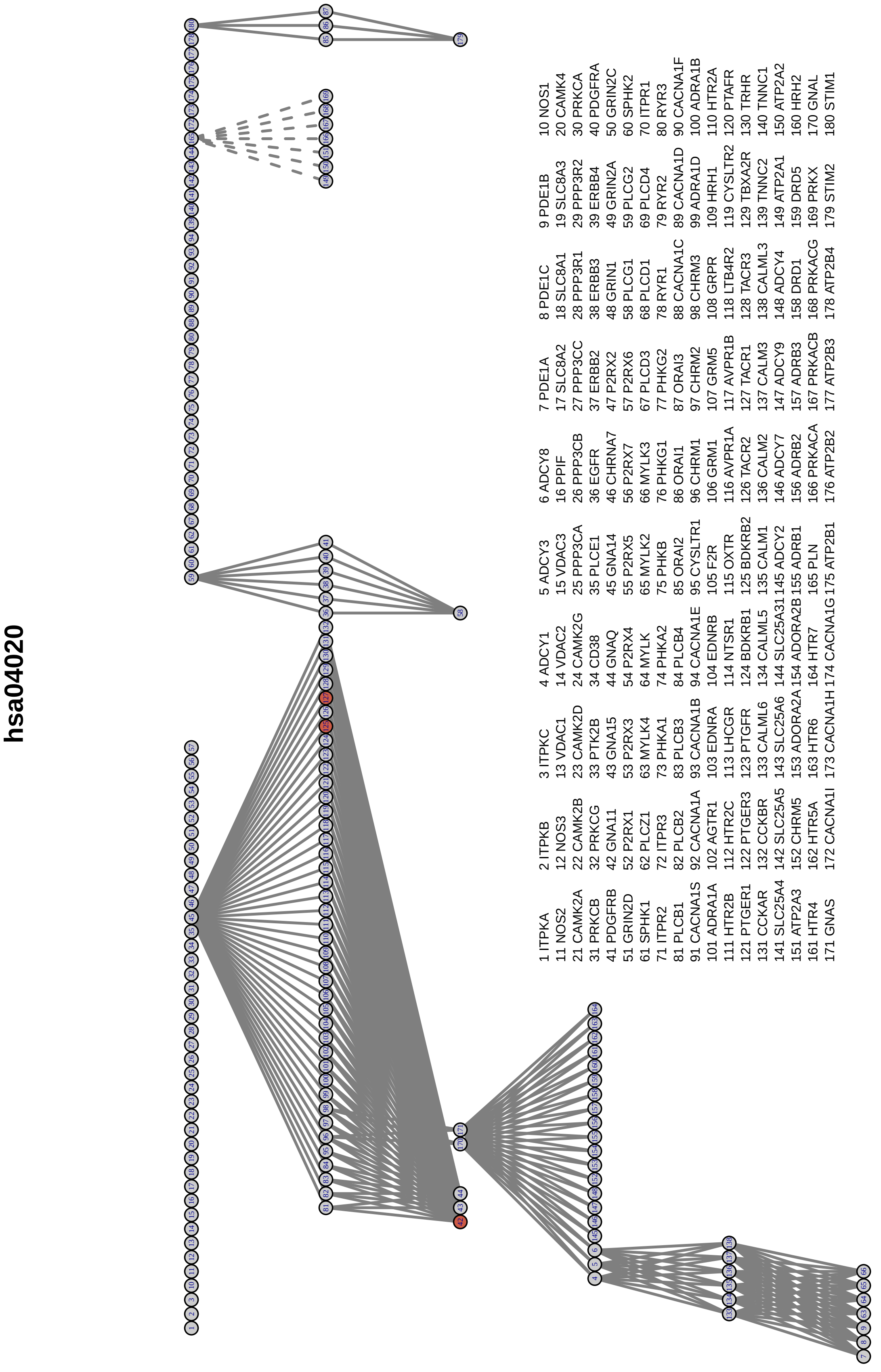

Figure 8: Network structure and placement of effect genes (red nodes) in the pathway hsa04020 used in simulations.

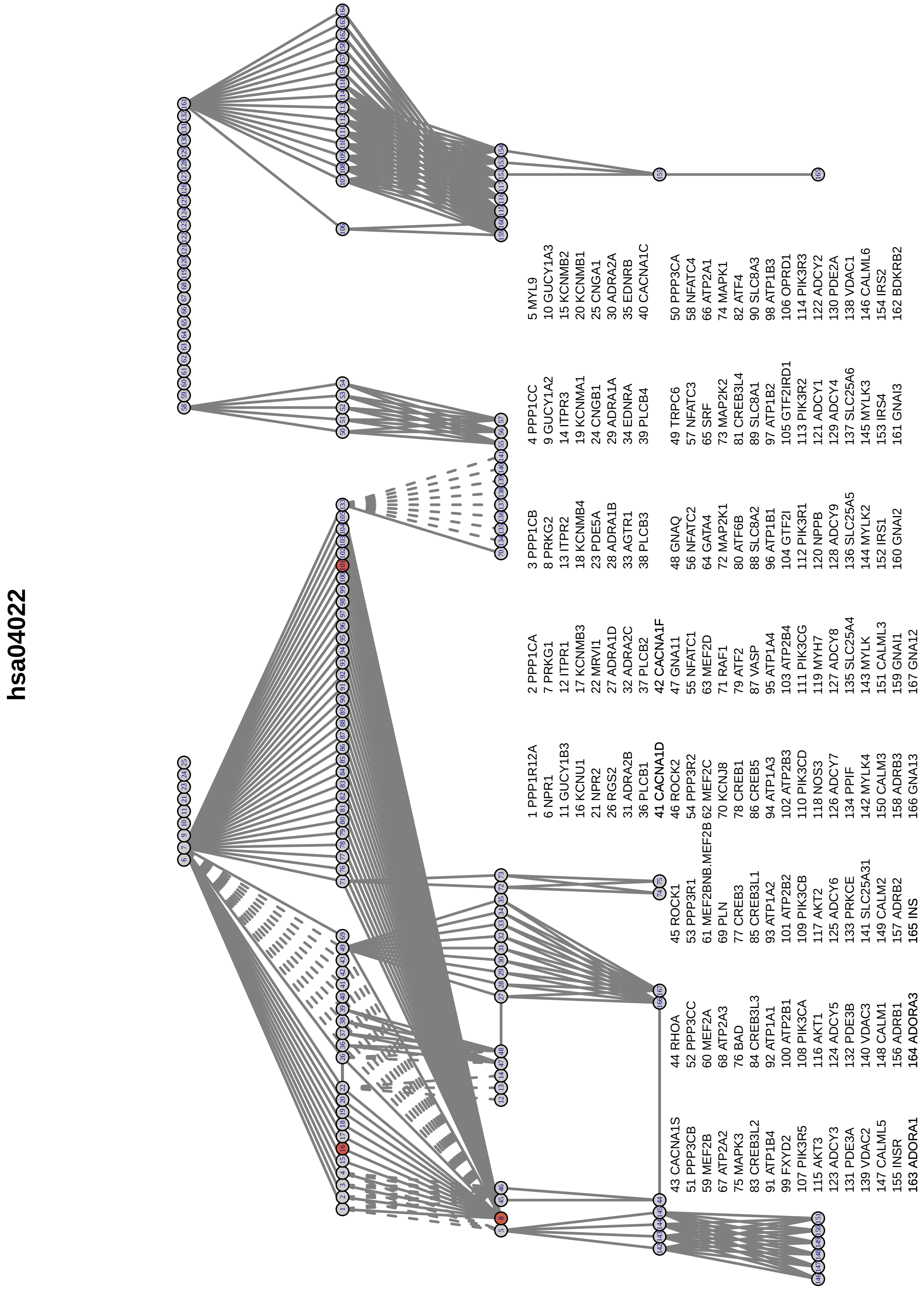

Figure 9: Network structure and placement of effect genes (red nodes) in the pathway hsa04022 used in simulations.

B Additional Results of Data Analyses

Figure 10 shows the out-of-bag risk for the 20-fold subsampling: The model is fitted 20 times on random subsets of the data and the (negative) Binomial likelihood is computed for the derived model on the new data (for each value of m_{stop}). Each of the gray lines is the out-of-bag risk for one model. The black line is the averaged risk for all 20 models. This estimates the goodness of fit, as measured by the likelihood, or better said the risk as measured by the negative likelihood. Essentially, we see how well the model would perform to predict the outcome for new data. The vertical dotted line indicates the optimal m_{stop} chosen on the dataset. The cross-validated risk for the lung cancer data shows that this data set seems to contain very little information as the risk almost imediately starts to increase. The optimal boosting iteration was chosen as $m_{\text{stop}} = 4$. The cross-validated risk for the rheumatoid athritis data shows that many updates were required to find the optimal model ($m_{\text{stop}} = 993$). It seems that this GWAS data set contains much more information on the disease status. The Receiver operating characteristic (ROC) curves of the two model for lung cancer and rheumatoid arthritis are depcited in Figure 11. These graphs display the overall prediction accuracy of the derived models.

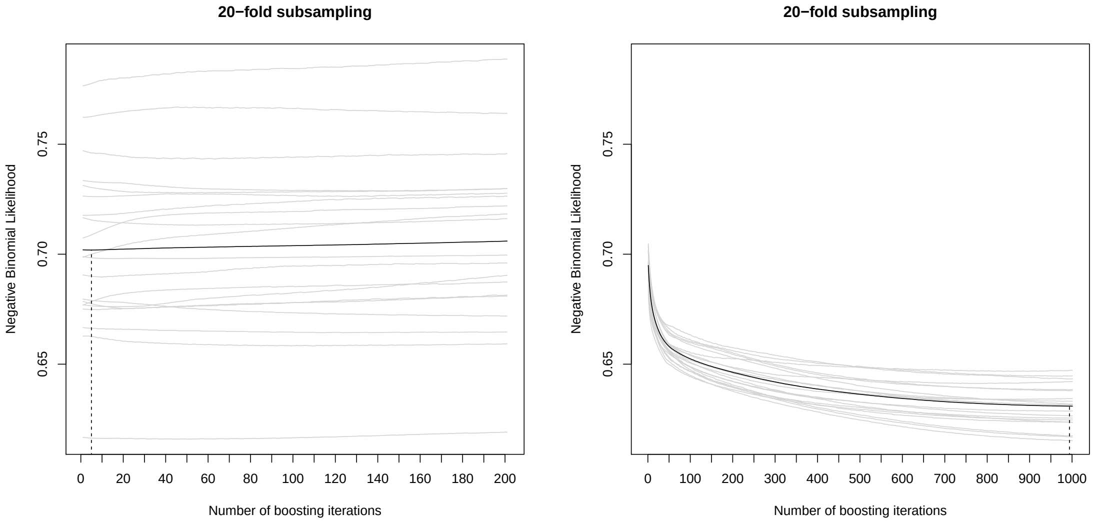

Figure 10: Cross-validated out-of-bag prediction accuracy for the lung cancer (left) and rheumatoid arthritis dataset (right).

Table 1 gives an overview the pathways used for the lung cancer data set together with the p-values derived via LKMT.

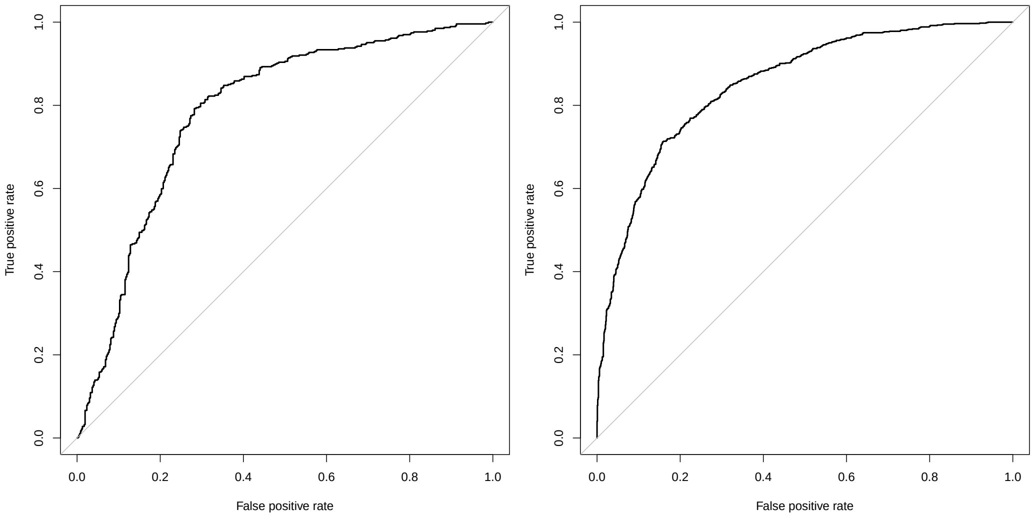

Figure 11: Receiver operating characteristic (ROC) curve depcting the prediction accuraccy of the boosted model for lung cancer (left) and for rheumatoid arthritis (right).

KEGG id	Name of Pathway	P-value
hsa05134	Legionellosis	0.0389
hsa05016	Huntington's disease	0.0446
hsa05323	Rheumatoid arthritis	0.0986
hsa05231	Choline metabolism in cancer	0.1232
hsa05210	Colorectal cancer	0.1421
hsa05169	Epstein-Barr virus infection	0.1464
hsa05220	Chronic myeloid leukemia	0.1698
hsa04940	Type I diabetes mellitus	0.1754
hsa05143	African trypanosomiasis	0.1758
hsa05014	Amyotrophic lateral sclerosis (ALS)	0.1800
hsa05205	Proteoglycans in cancer	0.1933
hsa05223	Non-small cell lung cancer	0.1991
hsa05144	Malaria	0.2080
hsa05211	Renal cell carcinoma	0.2274
hsa05332	Graft-versus-host disease	0.2590
hsa05214	Glioma	0.2653
hsa05212	Pancreatic cancer	0.3032
hsa05010	Alzheimer's disease	0.3177
hsa05031	Amphetamine addiction	0.3185
**hsa05020**	**Prion diseases**	0.3286
hsa05340	Primary immunodeficiency	0.3478
hsa05166	HTLV-I infection	0.3656
hsa05213	Endometrial cancer	0.4011
hsa04932	Non-alcoholic fatty liver disease (NAFLD)	0.4029
hsa05145	Toxoplasmosis	0.4054
hsa05218	Melanoma	0.4109
hsa05230	Central carbon metabolism in cancer	0.4262
hsa05330	Allograft rejection	0.4288
hsa04933	AGE-RAGE signaling pathway in diabetic complications	0.4297
hsa05206	MicroRNAs in cancer	0.4305
hsa05221	Acute myeloid leukemia	0.4315
hsa05219	Bladder cancer	0.4322
hsa05032	Morphine addiction	0.4411
hsa05133	Pertussis	0.4637
hsa05012	Parkinson's disease	0.4690
hsa05310	Asthma	0.4709
hsa05033	Nicotine addiction	0.4756

hsa05150	Staphylococcus aureus infection	0.4834
hsa05416	Viral myocarditis	0.5194
hsa05120	Epithelial cell signaling in Helicobacter pylori infection	0.5271
hsa05110	Vibrio cholerae infection	0.5287
hsa05161	Hepatitis B	0.5366
hsa05200	Pathways in cancer	0.5648
hsa04931	Insulin resistance	0.5697
hsa05217	Basal cell carcinoma	0.5736
hsa05030	Cocaine addiction	0.5852
hsa05215	Prostate cancer	0.5860
hsa05130	Pathogenic Escherichia coli infection	0.6437
hsa05204	Chemical carcinogenesis	0.6518
hsa05203	Viral carcinogenesis	0.6630
hsa05216	Thyroid cancer	0.6693
hsa05202	Transcriptional misregulation in cancer	0.6722
hsa05168	Herpes simplex infection	0.7000
hsa05131	Shigellosis	0.7154
hsa05100	Bacterial invasion of epithelial cells	0.7165
hsa05132	Salmonella infection	0.7292
hsa05320	Autoimmune thyroid disease	0.7341
hsa05152	Tuberculosis	0.7453
hsa05162	Measles	0.7702
hsa05222	Small-cell lung cancer	0.7793
hsa05140	Leishmaniasis	0.7971
hsa05142	Chagas disease (American trypanosomiasis)	0.8150
hsa05164	Influenza A	0.8419
hsa05322	Systemic lupus erythematosus	0.8594
hsa05146	Amoebiasis	0.8903
hsa05034	Alcoholism	0.8912
hsa04930	Type II diabetes mellitus	0.8960
hsa04950	Maturity onset diabetes of the young	0.9191
hsa05321	Inflammatory bowel disease (IBD)	0.9214
hsa05414	Dilated cardiomyopathy	0.9664
hsa05410	Hypertrophic cardiomyopathy (HCM)	0.9732
hsa05412	Arrhythmogenic right ventricular cardiomyopathy (ARVC)	0.9858
hsa05160	Hepatitis C	0.9863

Table 1: KEGG pathways in the Human Diseases class as downloaded in April 2016. Pathways are sorted according to p-value, derived from LKMT application on the lung cancer dataset, in ascending order. No pathways reached a significant p-value after Bonferroni correction are listed. The pathway selected by kernel boosting on this same dataset is marked in bold.

References

[1] Bühlmann P, Hothorn T. Boosting Algorithms: Regularization, Prediction and Model Fitting. Statistical Science. 2007;22:477–505.

[2] Mayr A, Binder H, Gefeller O, Schmid M. The Evolution of Boosting Algorithms - From Machine Learning to Statistical Modelling. Methods of Information in Medicine. 2014;53(6):419–427. Doi: 10.3414/ME13-01-0122.
